# Devices for minimally invasive liver parenchyma transection: the SICE (Italian Society of Endoscopic Surgery) Italian and International survey

**DOI:** 10.1007/s00464-025-11769-3

**Published:** 2025-06-16

**Authors:** Graziano Ceccarelli, Pasquale Avella, Edoardo Maria Muttillo, Maria Conticchio, Giovanni Domenico Tebala, Gaetano Piccolo, Lucia Romano, Riccardo Memeo, Aldo Rocca, LIVER-MIND (Minimally Invasive Surgery and pareNchyma transection Devices) Study Group, Davide Giovanni Grego, Roberto Lauro, Nicolas Pontarolo, Jacopo Andreuccetti, Rossella D’Alessio, Giusto Pignata, Giuseppe Calaciura, Nicola Cinardi, Riccardo Schillaci, Alessandro Mazzotta, Roberta Angelico, Luigi Eduardo Conte, Tommaso Maria Manzia, Marco Anania, Nicola Mancini, Marco Pazzona, Giuseppe Frazzetta, Antonio Picciurro, Annarita Libia, Marcello G. Spampinato, Cosimo Saviello, Giovanni Spiezio, Emanuele Pontecorvi, Vania Silvestri, Teresa Perra, Alberto Porcu, Alberto Oldani, Stefano Olmi, Alessandra Brescacin, Giampaolo Formisano, Matteo Barabino, Gaetano Piccolo, Andrea Barberis, Giovanni Tebala, Andrea Coratti, Giuseppe Giuliani, Francesco Guerra, Andrea Morini, Massimiliano Fabozzi, Maurizio Zizzo, Bruno Nardo, Daniele Paglione, Francesco Pata, Alexander Julianov, Azize Saroglu, Mattia Garancini, Fabrizio Romano, Mauro Alessandro Scotti, Andrea Quazzico, Stefano D’Ugo, Annarita Libia, Marcello Spampinato, Fabio Giannone, Fabrizio Panaro, Federico Sangiuolo, Giulia Lauteri, Federico Maggi, Luigi Masoni, Marcella Arru, Matteo Viti, Belkacem Acidi, Sang Thanh Nguyen, Michele Ammendola, Francesca Vescio, Tommaso Fontana, Flavio Milana, Fabio Procopio, Guido Torzilli, Giulio Argenio, Giorgio Ammerata, Giuseppe Currò, Giuseppe Sena, Curci Fabio Pio, Jose-Luis Beristain-Hernandez, Pietro Mezzatesta, Maria Cristina Saffioti, Andrea Belli, Francesco Izzo, Michele Ammendola, Francesca Vescio, Graziano Ceccarelli, Michele De Rosa, Fabio Rondelli, Andrea Tufo, Raffaele Galleano, Maurizio De Luca, Enrico Lodo, Dario Parini, Agostino Fernicola, Elio Jovine, Laura Mastrangelo, Christian Cotsoglou, Beatrice Torre, Mario Annecchiarico, Luigi Bonanni, Davide Chiappori, Federico Paniccia, Edoardo Saladino, Elisa Bertilone, Lorenzo Epis, Marco Filauro, Carolina Cecchi, Rosita De Vincenti, Massimo Fedi, Paolina Saullo, Massimiliano Fabozzi, Andrea Morini, Maurizio Zizzo, Marco Giordano, Gianmarco Palini, Luigi Veneroni, Gian Luca Grazi, Antonio Taddei, Luca Tirloni, Gennaro Mazzarella, Giulia Bacchiocchi, Edoardo Baldini, Alberto Brolese, Francesca Notte, Stefan Patauner, Giovanni Scotton, Riccardo Caruso, Yolanda Quijano, Emilio Vicente, Stefano Cantafio, Egidio Miranda, Federica Maffeis, Paolo Ubiali, Jaqueline Velkoski, Francesco Tandoi, Alessandro Capozucco, Alessandro Liguori, Alessandro Puzziello, Mariafelicia Valeriani, Chiara Bettini, Martina Fricano, Sarah Molfino, Giuseppe Evola, Luigi Piazza, Marco Vacante, Alessandro Cucchetti, Giorgio Ercolani, Giuliano La Barba, Andrea Benedetti Cacciaguerra, Federico Mocchegiani, Marco Vivarelli, Giammauro Berardi, Giuseppe Ettorre, Alessandro Anselmo, Silvio Caringi, Leandro Siragusa, Paolo Bianco, Fulvio Calise, Salvatore Spiezia, Alberto Patriti, Filippo Petrelli, Luca Viganò, Antonio Giuliani, Stefano Berti, Daniele Celi, Valentina Marchese, Edoardo Maria Muttillo, Paolo Mercantini, Andrea Scarinci, Celia Caula, Margarida Casellas Robert, Santiago Lopez Ben, Tommaso Campagnaro, Mario De Bellis, Andrea Ruzzenente, Roberto Montalti, Gianluca Rompianesi, Roberto Troisi, Francesco Orlando, Giovanni Vennarecci, Francesco Ardito, Francesco Razionale, Benedetto Ielpo, Luca Risi

**Affiliations:** 1 Department of General Surgery, San Giovanni Battista” Hospital, USL Umbria 2, Foligno, Perugia, Italy; 2 Department of Medicine and Health Science “V. Tiberio”, University of Molise, Campobasso, Italy; 3 Hepatobiliary and Pancreatic Surgery Unit, Pineta Grande Hospital, Castel Volturno, Caserta, Italy; 4 Department of Clinical Medicine and Surgery, University of Naples “Federico II”, Naples, Italy; 5 Department of Medical Surgical Science and Translational Medicine, Sant’Andrea University Hospital, Sapienza University of Rome, Rome, Italy; 6 Unit of Hepato-Pancreato-Biliary Surgery, Miulli Hospital, Acquaviva delle Fonti, Bari, Italy; 7 Department of Digestive and Emergency Surgery, S. Maria Hospital Trust, Terni, Italy; 8 Department of Health Sciences (DISS), General Surgery Unit, University of Milan San Paolo Hospital, Milan, Italy; 9 Department of Biotechnological and Applied Clinical Sciences, University of L’Aquila, L’Aquila, Italy

**Keywords:** Liver surgery, Robotic liver surgery, Laparoscopic liver surgery, High energy device, Minimally invasive surgery, Liver parenchyma, Indocyanine green fluorescence, High volume, Medium volume, Low volume

## Abstract

**Backgrounds:**

Minimally Invasive Liver Surgery (MILS), encompassing laparoscopic (L-MILS) and robotic (R-MILS) approaches, has revolutionized liver surgery, offering reduced morbidity, shorter hospital stays, and improved outcomes while maintaining oncological efficacy. Despite the widespread use of L-MILS, parenchyma liver transection techniques and devices remain debated. This study investigates the adoption of transection devices (TDs) in MILS among 86 hospitals, focusing on surgical practices, device utilization, and outcomes.

**Methods:**

The Italian Society of Endoscopic Surgery (SICE) endorsed a cross-sectional internet-based survey targeting general and Hepato-Pancreato-Biliary surgeons.

**Results:**

Responses from 86 centers revealed that 77% of institutions is available a robotic platform, with an adoption rate of 87.50% in high-volume centers. L-MILS remains the predominant technique for liver resections, also in case of major hepatectomies, although R-MILS is increasingly utilized. For minor L-MILS, more than 50% of respondents use ultrasonic shears and electrosurgical pencil and advanced bipolar devices, while about 40% of surgeons adopt Cavitronic Ultrasonic Surgical Aspirator (CUSA) in major resections. R-MILS procedures predominantly used Maryland bipolar forceps and vessel sealers, with hybrid techniques (30%) integrating laparoscopic devices (e.g., CUSA) to address robotic device limitations.

**Conclusion:**

The minimally invasive approach to liver parenchymal transection is a key component of this surgical procedure. For major hepatectomies, the CUSA device remains the most effective tool, whereas ultrasonic shears, electrosurgical pencil, and advanced bipolar devices are more suited for minor resections. Despite limited access to specialized instruments, R-MILS achieves favorable outcomes in liver transection by employing the crash-clamp technique or hybrid strategies.

**Supplementary Information:**

The online version contains supplementary material available at 10.1007/s00464-025-11769-3.

Liver surgery is considered a high morbidity and mortality surgery, particularly due to the challenges of controlling bleeding and managing complex liver anatomy [1–5].

Laparoscopic and Robotic Minimally Invasive Liver Surgery (L-MILS and R-MILS) has changed Hepato-Pancreato-Biliary (HPB) surgery landscapes, reducing the length of stay and postoperative complications and improving patient outcomes with superimposable results compared to traditional open surgery [6–13].

Contemporarily, the evolution of surgical techniques in MILS has allowed for greater precision in liver resections, enhancing imaging techniques such as Intraoperative Ultrasound (IOUS) and Indocyanine Green (ICG) fluorescence and refining sophisticated transection devices [14–17].

As the field continues to evolve, understanding the adoption and impact of these advanced techniques across various medical centers is crucial to optimize patient care and guiding future innovations in liver surgery.

The widespread adoption of robotic platforms over the past decade has opened new horizons, offering high-definition 3D visualization, advanced articulating EndoWrist technology, microsurgical precision, and enhanced capabilities to manage complex conditions.

However, one of the major concerns about R-MILS is linked to the limited choice of transection devices dedicated to liver surgery widely used in open and laparoscopic fields [18]. Despite this, R-MILS has increased in the western and eastern countries [12, 19–26].

Taking into account the above considerations, this survey aims to evaluate the current state of MILS, primarily focusing on the adoption of transection devices.

The survey also provides some data on center volumes and techniques and outcomes, so our study seeks to identify trends, challenges, and outcomes associated with different surgical approaches, providing a robust foundation for future clinical practice and consensus.

## Materials and methods

### Survey

The Italian Society of Endoscopic Surgery (SICE) endorsed an internet-based cross-sectional survey to investigate the percentage of MILS across public and private international hospitals and to evaluate the use of several Transection Devices (TDs) for liver resection according to parenchyma features (healthy, steatosis, or cirrhosis), hospital volumes (high, medium, or low) and type of L-MILS and R-MILS.

Google Forms Survey® (Google; Mountain View, CA, USA) was used to disseminate our survey and to collect individual informed consent. Thanks to an email, a brief study purpose was sent to SICE members, and their participation remained voluntary. Recipients received two reminders.

The survey was performed according to the Checklist for Reporting Results of Internet E-Surveys (CHERRIES) [27].

The Steering Committee (G.C., R.M.), the Methodologist (P.A.), and the Expert Committee (P.A., E.M.M., M.C., G.T., G.P., L.R., A.R.) developed 60 questions after remote discussions about MILS and TDs currently used worldwide. Before going live, the questionnaire was put through a preliminary beta testing phase to make sure it was user-friendly, relevant, and understandable.

The final survey consisted of 44 questions divided into three sections (Appendix 1, Table 1). Both closed-ended (38/44, 86%) and open-questions (6/44, 14%) were included.

**Table 1:** Type of different transection devices commonly used in laparoscopic and robotic liver resection

Type of transection devices	
Laparoscopic devices	Robotic devices*
**Laparoscopic monopolar hook**	**Robotic Monopolar hook**
**Laparoscopic monopolar scissor**	**Robotic Monopolar scissor**
**Bipolar Cautery**	**Robotic Maryland bipolar**
**Advanced Bipolar devices** - LigaSure™ (Medtronic, USA) - ENSEAL (Ethicon Endo-Surgery, USA)	**Advanced Bipolar devices** - Vessel Sealer Extend (Intuitive Surgical, USA) - SynchroSeal (Intuitive Surgical, USA)
**Ultrasonic shears** - Lotus Torsional® (BOWA Medical, UK) - Harmonic® (Ethicon Endo-Surgery, USA) - Sonicision™ (Medtronic, USA) - HARMONIC ACE® + 7 Shears (Ethicon Endo-Surgery, USA) - Ultracision® (Covidien, USA) - SonoSurg® (Olympus, Japan)	**Ultrasonic shears** - HARMONIC ACE® (Intuitive Surgical, USA)
**Advanced bipolar and ultrasonic devices** - THUNDERBEAT® (Olympus, Japan)	**Ultrasonic + Radiofrequency**
**Staplers** - Endo GIA™ Universal (Medtronic, USA) - Endo GIA™ Ultra Universal (Medtronic, USA) - Endo GIA™ with Tri-Staple™ Technology (Medtronic, USA) - ECHELON FLEX™ Powered Plus Stapler (Ethicon Endo-Surgery, USA) - ECHELON FLEX™ GST System (Ethicon Endo-Surgery, USA) - Signia™ Stapling System (Medtronic, USA)	**Staplers** - SureForm™ (Intuitive Surgical, USA)
**Monopolar or Bipolar Radiofrequency needle** - HabibTM 4X (LH4X, Rita, USA), - Aquamantys™ (Medtronic, USA)	
**Saline-linked radiofrequency dissector** - TissueLink medical’s DS3.5 (Dover, NH)	
**Cavitronic Ultrasonic Surgical Aspirator** - CUSA EXcel® (Integra, Ireland) - Sonoca (Söring GmbH, Germany) - SonoSurg® Ultrasonic Aspirator (Olympus, Japan)	
**Water-jet** - Helix Hydro-Jet (Erbe, Tubingen, Germany)	
**Precoagulation** - Radiotherapy-assisted devices	
**Laparoscopic Argon beam coagulators**	**Robotic Argon beam coagulators**
**Polymeric clips** - Hem-o-lok® (Weck Surgical Instruments, Teleflex Medical, USA)	**Polymeric clips** - Hem-o-lok® (Weck Surgical Instruments, Teleflex Medical, USA)
	**Extended grasper**

*Nowadays, all robotic instruments are commercialized by Intuitive

The first two sections covered general inquiries concerning the respondents’ baseline characteristics, their nation and continent of practice, their type of subspecialty and years of training, and the hospital organization. The last section covered specific types of clinical practice according to MILS approaches and liver parenchyma features.

The survey has been sent to respondents between July and August 2024.

General and demographics data preempt dedicated liver surgery questions including years of experience, volume (low < 20 resections/year, medium 20–50 resections/year, high > 50 resections/year), type (public, academic, private) of center, and available equipment for MILS.

Major L- and R-MILS resections were defined as ≥ 3 Counaid liver segments (anatomically major) or 7, 8, 4a, and 1 segment resections (technically major) [28]. Difficult liver resections were defined according to IWATE score: Operative levels > 6 were reported as challenging procedures [29].

### Participants

General and HPB surgeons, including surgical trainees and fellows, consultant/attending surgeons, researchers/professors, and directors, attended our survey.

Hospitals from North and South America, Europe, Asia, Africa, and Oceania were featured.

We excluded survey participants who provided less than 60% of responses.

### Transection devices

Our survey was developed to gather quantitative data on different TDs used in L- and R-MILS according to anatomical/non-anatomical, major/minor resections, and liver parenchyma texture.

A structured questionnaire was constructed, incorporating multiple-choice and Likert-scale questions to facilitate standardized responses. The various TDs indicated in our survey were reported in Table 1.

Usage percentages were divided into five distinctive categories: 0%, < 25%, 25–50%, 51–75%, and > 75%. This segmentation was intended to capture both non-use and varying degrees of device adoption, providing a comprehensive overview of the current practice patterns in parenchymal transections.

### Statistical methods

Data was analyzed with IBM Statistical Package for the Social Sciences (IBM SPSS®). Quantitative variables were reported as mean ± Standard Deviation (SD) or median and interquartile range (IQR). Frequencies and percentages were used to depict the categorical data.

The chi-square test (χ^2^) or Fisher’s exact test was performed to compare qualitative variables, while the T-test was performed to analyze normal distributed variables.

A two-tailed *p* value < 0.05 was recognized as statistically significant. Continuous data that were not normally distributed were shown as 95% confidence intervals (95%CI) or as medians and interquartile ranges (IQRs).

Variables were stratified among the 3 different volume centers—High Volume (HV, ≥ 50 liver resection/year), Medium Volume (MV, 20–49 liver resections/year), and Low Volume (LV < 20 liver resections/year)—and center type (private or public), compared using contingency tables, and analyzed using the Chi-square test for categorical or one-way analyses of variance (ANOVA) for continuous variables, respectively.

Sensitivity analysis was done for center type as well as the total yearly volume of liver surgeries.

### Ethical issue

For this research, which involved a non-intrusive, anonymized web-based survey, no formal ethical approval was necessary.

## Results

### Baseline characteristics of respondents

Our survey received a total of 86 responses that included 178 surgeons and 86 centers from 7 countries (Table 2). Europe represents the most popular country (78.1%).

**Table 2:** Characteristics of centers involved in SICE survey according to MILS volumes

	**All**	**LV**	**MV**	**HV**	***p*** **value**
Number of centers; (%)	86 (100)	27 (31.4)	35 (40.7)	24 (27.9)	
MILS procedures, yes; *n*. (%)	85 (98.8)	26 (96.3)	35 (100)	24 (100)	*1.0000*
MILS type, *n*. (% - Laparoscopy - Robot - Both	33 (38.4) 9 (10.5) 48 (55.9)	17 (63) 2 (7.4) 7 (25.9)	13 (37) 5 (14.2) 22 (62.8)	3 (12.5) 2 (8.3) 19 (79.2)	*0.0021*
Type of robotic devices available, n. (%) - Da Vinci X, Intuitive Surgical - Da Vinci Xi, Intuitive Surgical - Hugo RAS, Medtronic*	4 (4.6) 60 (69.8) 2 (2.3)	2 (7.4) 14 (51.8) 0 (0)	1 (2.9) 25 (71.4) 1 (2.9)	1 (4.2) 21 (87.5) 1 (4.2)	*0.7100*
L-MILS per year, n. (%) - < 20% - 21–30% - 31–50 - > 50% - NA	35 (40.7) 19 (22.1) 16 (18.6) 13 (15.1) 3 (3.5)	15 (55.6) 6 (22.2) 2 (7.4) 1 (3.7) 3 (11.1)	12 (34.3) 10 (28.6) 9 (25.7) 4 (11.4) 0 (0)	8 (33.3) 3 (12.5) 5 (20.8) 8 (33.3) 0 (0)	*0.0069*
R-MILS per year, n. (%) - < 20% - 21–30% - 31–50 - > 50% - NA	40 (46.5) 12 (14) 10 (11.6) 9 (10.5) 15 (17.4)	19 (73.1) 1 (3.8) 0 (0) 1 (3.8) 6 (22.3)	12 (34.3) 6 (17.1) 3 (8.6) 7 (20) 7 (20)	9 (37.5) 5 (20.8) 7 (29.2) 1 (4.2) 2 (8.3)	*0.0019*

Italics indicate statistical significance

*Hugo Robotic Assisted Surgery, Medtronic, is still not available for MILS

*MILS* Minimally Invasive Liver Surgery, *L-MILS* Laparoscopic–Minimally Invasive Liver Surgery, *R-MILS* Robotic–Minimally Invasive Liver Surgery, *NA* Not Available

The median age of responding surgeons was 45.29 ± 10.63 years (range 27–68 years). Participating were HPB surgeons who also trained in general (34.4%), colorectal (32.2%), and emergency surgery (7.9%). Overall, they had a duration of surgical activity lasting 10.7 years. Responders mainly came from public central hospitals (73%). MV centers represented the majority of respondents (40.7%), while LV were represented by 31.4% of responders followed by HV centers (27.9%) (*p* = 0.579).

**Fig. 1:**
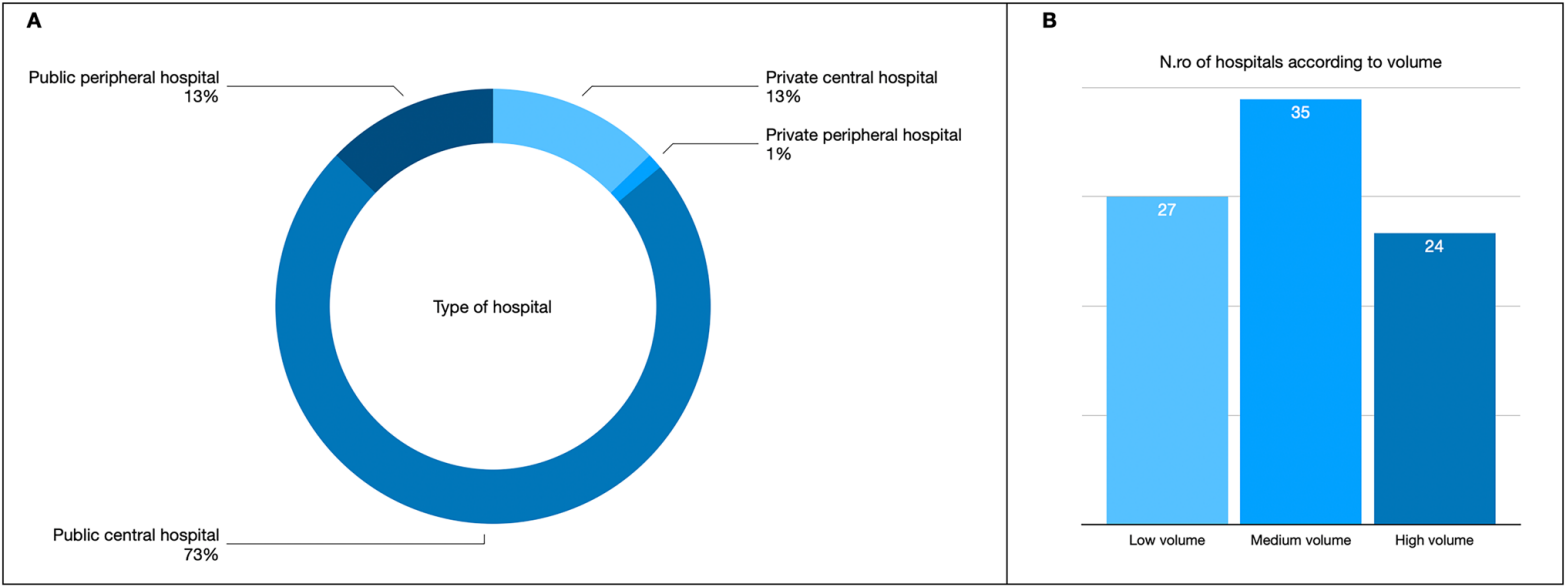
**A**, Distribution of hospital types by Central *vs.* Peripheral and Public *vs.* Private policy (*p* value = < 0.0001); **B**, Number of centers according to Minimally Invasive Liver Surgery volume (*p* value = 0.579)

Figure 1 summarized distribution of hospital types according to public and private policy and volume centers.

### Minimally invasive liver surgery experience and availability

The number of MILS procedures performed by year are listed in Table 2. Although L-MILS are uniformly distributed across respondents’ centers, R-MILS is more frequently available at HV (*p* = 0.0021).

The 77% of respondents declared that a robotic device is available in their institution. Therefore, analyzing robotic availability (Table 2), it is possible to conclude that R-MILS is not performed in all centers while in 55% of cases it is used in all general surgery procedures.

While 79% and 62% of HV and MV centers indicated performing both L- and R-MILS, only 25% of LV declared whether laparoscopic or robotic devices are available in their center.

Across all centers involved in our survey, in case of challenging procedures (IWATE ≥ 6) or major hepatectomies (≥ 3 liver segments), L-MILS represents the preferred technique (44.9%), followed by R-MILS (38.5%) and both approaches (16.6%).

### TDs equipment

**Fig. 2:**
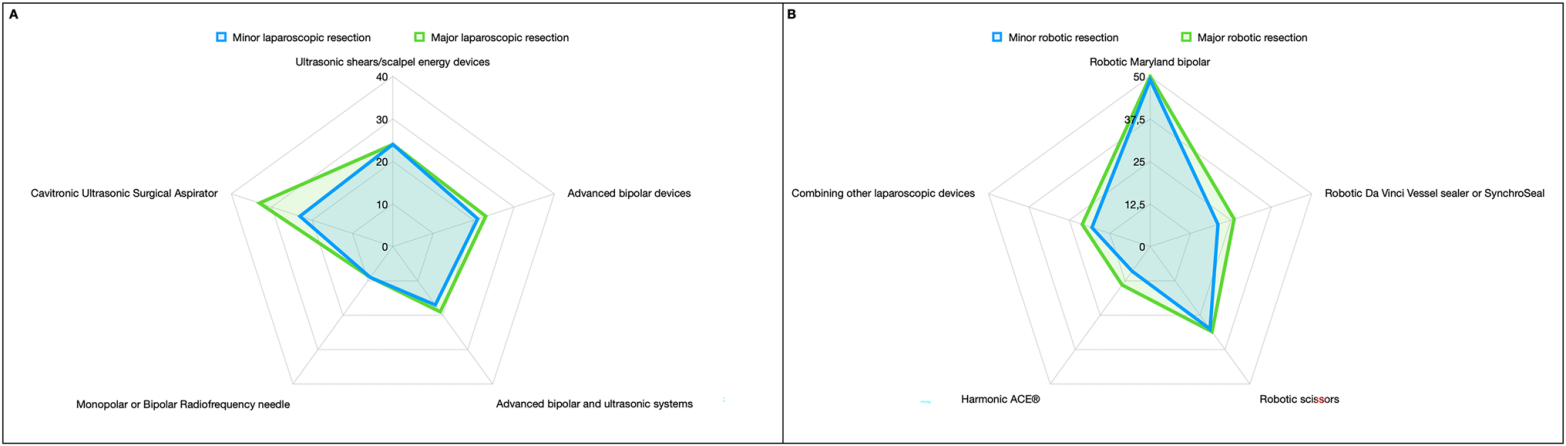
Radar plots comparing the utilization of surgical devices in different procedures. **A**, Minor versus major laparoscopic resections: Utilization levels of ultrasonic shears/electrosurgical pencil devices, advanced bipolar devices, monopolar or bipolar radiofrequency needles, advanced bipolar and ultrasonic systems, and Cavitronic Ultrasonic Surgical Aspirators. **B**, Minor versus major robotic resections: Utilization levels of Robotic Maryland Bipolar, Da Vinci Vessel sealer or SynchroSeal, robotic scissors, Harmonic ACE®, and combinations of other laparoscopic devices

Figure 2 clarify the usage distribution of TDs in L- and R-MILS approaches, segmented by minor or major resections.

Among the surveyed institutions, for minor L-MILS, Harmonic® (Ethicon Endo-Surgery, USA) and LigaSure™ (Medtronic, USA) emerged as the most used device. Cavitronic Ultrasonic Surgical Aspirator (CUSA) followed closely, being the primary choice in more than 25% of centers. Additionally, less than 20% of centers predominantly utilized the THUNDERBEAT® (Olympus, Japan), highlighting its dual energy capability combining ultrasonic and advanced bipolar energy. Statistical analysis does reveal a significant variation in device preference among centers (*p* = 0.0947).

In the context of major hepatic surgery, the analysis of device utilization across 86 centers reveals distinct preferences. The CUSAs were the most employed devices (38%), Harmonic® (Ethicon Endo-Surgery, USA) followed closely (27%) being the primary choice in > 25% of centers, while LigaSure™ (Medtronic, USA) was preferred in about 25% of centers. Additionally, as reported in minor L-MILS, the THUNDERBEAT® (Olympus, Japan) was used in 20% of centers. Statistical analysis revealed a significant variation in device preference among centers (*p* = 0.007).

Although CUSAs use is more frequent in major hepatic resections, no statistically significant difference in its preference among centers was observed between major and minor MILS.

For minor and major R-MILS, the Maryland bipolar forceps is the most used instrument, adopted by more than 50% of surgeons, followed by bipolar scissors and vessel sealer. Additionally, 18 out of 86 (20.93%) centers employ a hybrid approach, integrating a laparoscopic instrument to perform a robotic resection to overcome the limited availability of robotic devices. This hybrid approach underscores the adaptability of surgeons in optimizing resources to perform complex resections effectively. The choice for a hybrid technique appears to be strongly linked to the dissatisfaction expressed by 35% of robotic respondent surgeons, who report limitations with the currently available instrumentation.

Saline-Linked Devices, Water Jet Devices, Argon Beam Coagulators and Precoagulation Devices exhibit the highest non-use, indicating that these devices are rarely chosen for minor and major parenchymal transections.

### Parenchyma transection

**Fig. 3:**
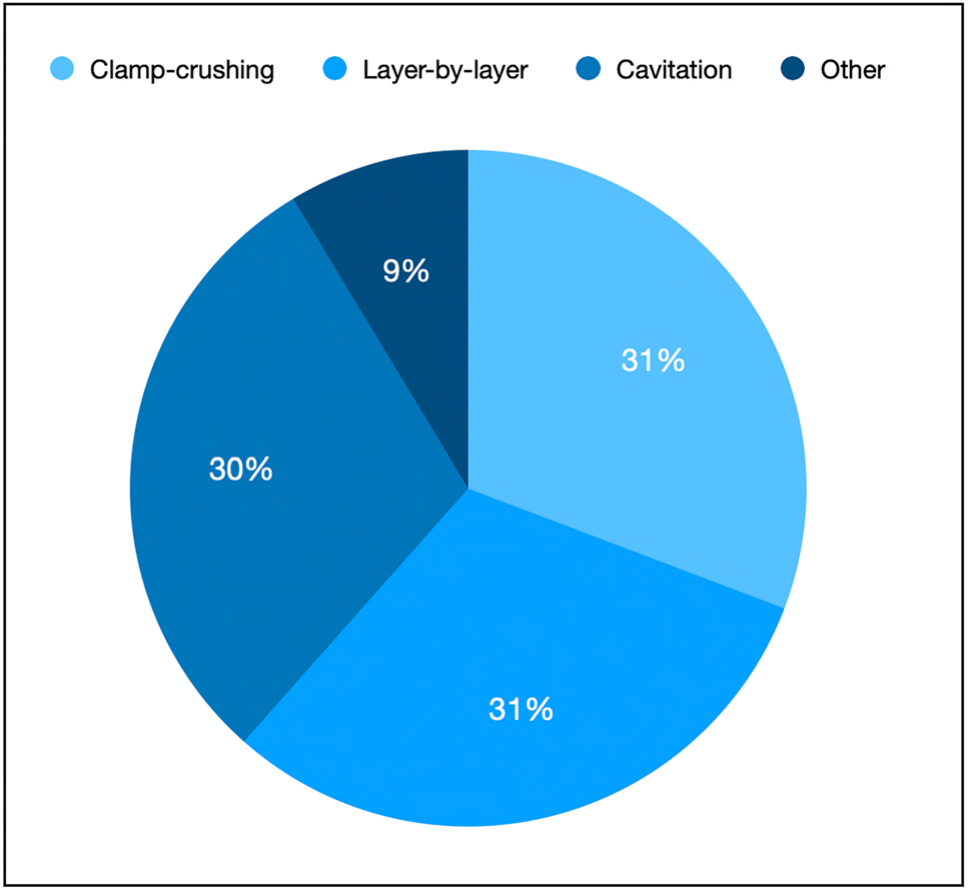
The dominance of traditional techniques: Clamp-crush technique and layer-by-layer transection constitute 60% of the reported methods. The hybrid approaches, thanks to laparoscopic CUSA devices, play a notable role, reflecting variability in surgical practices

The pie chart (Fig. 3) presents data on the techniques used for robotic liver dissection in both minor and major resections. Clamp-crush technique (32%) and Layer-by-layer Transection (32%) are the most employed pure robotic techniques. Cavitation, using laparoscopic devices, is used in 31% of centers.

The adoption of staplers is about 10%, potentially reserved for specific clinical scenarios or anatomical considerations.

This distribution underscores the diversity in approaches to robotic liver dissection and reflects the balance between traditional and innovative techniques in this field.

### Liver parenchyma texture

A survey assessed surgeons’ approaches to handling cirrhotic liver parenchyma during transection. Among 86 centers, 31.4% reported modifying their techniques for cirrhotic livers: CUSAs were the most used instruments (54.8%), followed by bipolar devices (47.6%) and a combination of ultrasonic and radiofrequency devices (42.9%).

### Vascular control

In our survey, two questions aimed at understanding surgeons’ practices related to vascular control during liver surgery. The first question investigated the routine preparation of inflow vascular control before liver transection, whether for minor or major surgeries. Out of 86 respondents, the majority (74.1%) indicated a preparation of inflow vascular control. A smaller proportion (21.2%) reported only in selected cases, while 4.7% of respondents declared “no-planning technique.”

The second question explored the Pringle maneuver use or selected vascular control during liver resection. Among 86 respondents, an overwhelming majority (98.9%) confirmed performing the Pringle maneuver.

### Hemostatic technique and topical sealants

Regarding hemostasis, the survey focused on vessel management and topical sealants. For major vascular and biliary branches, the most popular (42/86, 48.8%) technique across all centers was represented by the Polymer Ligation Clip System (e.g., Hem-o-lok™ clips, Weck, USA) application, followed by titanium clips (26/86, 30.2%) and knot-type ligation (16/86, 18.6%). However, 1 (2.9%) of 35 MV centers reported using radiofrequency for vessel hemostasis control.

More than 90% of centers declared that applying topical sealants to the liver surface: Fibrinogen-based sealant (*e.g.,* Tachosil) was preferred in about 60% of centers, while 25% of surgeons reported a collagen-based sealant application (e.g., Tissucol). One center routinely applied oxidized regenerated cellulose (e.g., SURGICEL™ Absorbable Hemostat).

### Intraoperative ultrasound and indocyanine green fluorescence

All involved centers performed IntraOperative UltraSound (IOUS), using laparoscopic or dedicated robotic probes to schedule surgical liver resection.

There is no statistically significant difference in the selective use of ICG across surgical volumes. Nevertheless, HV showed a higher percentage of routine application than MV and LV (79.2 *vs.* 42.8 *vs.* 50%, respectively).

## Discussion

This survey on minimally invasive surgery (laparoscopic and robotic) reported and analyzed the diffusion and indications in the use of TDs in liver surgery, focusing on their spread, availability, surgical confidences, and transection techniques used according to minor and major hepatectomies. Furthermore, it also provided data on center volume techniques and outcomes, identifying trends, challenges, and outcomes associated with different surgical approaches and providing a robust foundation for future clinical practice and consensus.

The most important challenge during minimally invasive liver parenchyma transection is to obtain the same intra- and post-operative outcomes of conventional open surgery, in terms of minimum blood loss, adequate surgical margin clearance for malignancy diseases, operative time, parenchyma sparing if requested, low postoperative bile leak.

To achieve these effective results, several dissection devices have been developed during the last 20 years (Table 1), as demonstrated in the successive consensus held in Louisville, Morioka, and Southampton [30–32].

Among TDs most favored by surgeons are CUSA devices [33], while decline in the utilization rate of the Habib device has been observed. The laparoscopic Habib 4X is a bipolar device which consists of an array of four electrodes. The electrodes are made of stainless steel and covered with a non-stick coating (Tomlinson Tube & Instrument Ltd., Warwickshire, UK). The four needles are arranged in a 2 × 2 array with the two pairs of needles connected and each pair is connected to a single terminal of a bipolar radiofrequency generator (Generator 1500x, RITA Medical Systems Inc., California, USA) [34].

The development of advanced dissection instruments has progressively rendered hand-assisted laparoscopy out-of-date, despite the advantages of maintaining a minimally invasive approach while using the hand for improved retraction, facilitating optimal exposure, and enabling rapid control of significant bleeding when necessary [35].

The widespread of R-MILS has deeply change the minimally invasive scenario, offering the chance to have a 3D immersive vision, a 360° EndoWrist degree of movements, and an excellent use of standard bipolar instruments. The findings of the bibliometric analysis conducted in 2023 by Zeng et al. highlight that the development of robotic surgery in the hepatobiliary field remains a subject of ongoing discourse [36]. Notably, between 2003 and 2022, a total of 685 studies were published in the literature. However, a significant proportion—454 articles, accounting for more than 60%—were published within the last 5 years (2018–2022), underscoring the recent surge in interest and advancements in this area.

On the other side, the lack of standardized R-MILS, and the absence of dedicated robotic TDs (particularly CUSAs) represents a challenge for HPB surgeons, encouraging the adoption of several techniques included the hybrid technique “Robo-Lap approach” integrates the use of the robotic platform—specifically robotic bipolar forceps for coagulation and scissors—with a laparoscopic dissector operated directly by the surgeon at the bedside [37]. Indeed, the “two-surgeon technique” involves the presence of two experienced HPB surgeons in the operating room. In this approach, the primary surgeon performs the parenchymal transection using the robotic platform, while the second surgeon assists at the operating table using laparoscopic instruments. In contrast, the “one-surgeon technique” is defined by a single HPB surgeon operating from the robotic console, performing a pure robotic procedure, while a general surgeon assists with tissue exposure, suction, and irrigation.

To overcome the R-MILS limitations, several techniques were described in literature.

At the beginning of parenchyma transection, the liver capsule is usually divided thanks to monopolar energy to demarcate the planned transection line, following verification of navigation tools within the console, such as IOUS, ICG dye staining, or consultation of a 3D model [38].

Subsequently, one of the commonly used techniques for parenchyma transection is represented by “microfracture-coagulation method” also known as the “clamp-crush technique,” simulating the open kellyclasia [38, 39]. This “cold dissection” approach primarily involves the simultaneous use of EndoWrist bipolar Maryland forceps and EndoWrist monopolar curved scissors [38]. The technique is facilitated by the assistant using an irrigator through a trocar to keep the tip of the bipolar instrument moist [38]. The outcome of mechanical hepatocyte fragmentation allows the identification of biliary and vascular elements: Structures with a diameter of ≤ 5 mm can typically be coagulated with bipolar energy, while larger structures generally require the application of clips, such as titanium or Hem-o-lok. Larger hepatic veins are typically managed with a laparoscopic or robotic vascular stapler [38].

Kajiwara et al*.* described a variant to “clamp-crush technique” adding a saline-linked bipolar [40]. A continuous drip of saline solution (1–2 ml/min) is applied to the tips of the bipolar forceps by the assistant surgeon using a ball-tipped electrocautery device. This prevents the buildup of necrotic or coagulated liver tissue, eliminating the need for cleaning the forceps due to eschar or char formation. Ensuring proper moisture levels minimizes procedural interruptions and reduces the frequency of breaks during hepatic transection. Authors defined this method as “non-stick liver parenchymal transection.”

Birgin et al*.* introduced an alternative fully robotic technique referred to as the “scissors hepatectomy technique” [41]. This method achieves liver parenchymal transection exclusively through the combined use of monopolar scissors and bipolar forceps. Notably, the approach eliminates reliance on traditional energy-based devices, instead emphasizing precise mechanical dissection and coagulation provided by these instruments.

Another robotic technique is the “onion veil (layer by layer) technique” generally using the robotic harmonic scalpel (not articulated instrument) [42], while the “burn and push technique” reckon on liver parenchyma coagulation and then separated by a gentle lateral push using the Monopolar curved scissors. Vessel or Glissonean pedicle are instead managed by the clamp‐crush technique with the Maryland bipolar forceps [43].

Among the described techniques in the literature, the following should also be highlighted. The silicone band or others slings techniques are usually used during L- and R-MILS transection for tractions, hanging or separation of liver parenchyma [44]. Saline-Linked Electrocautery Combined With Wet Oxidized Cellulose (SLiC-WOC) approach combines two different hemostatic techniques achieving an efficient and reliable hemostasis [45].

Single Incision Laparoscopic or Robotic Surgery approaches represent the latest evolutions in minimal invasive surgery and has been increasingly utilized in abdominal surgery. The evidence on its use in liver and pancreatic resectional surgery is scarce and limited to published case reports and small case series [46].

We also analyzed the use of near infrared fluorescence imaging with ICG in MILS. The technique may be used for tumor identification with a previous dye injection of at least 24 h (2.5 mg of ICG injected intravenously), tumor margins identification (to achieve negative R0 margin of resection), or used as guide for anatomical liver resections by a demarcation line after portal branch clamping (for anatomical resections) [14]. Its use in laparoscopy and robotic was investigated in our survey. We have to consider that this technology is not always present in every operating room and only recently adopted in the new laparoscopic equipment. In robotic surgery a simple switch to ICG vision allow the negative and positive parenchyma staining view [47]. Associated to intraoperative ultrasound may represent the best way to manage liver tumor [48].

Cost analysis and cost-effectiveness related to the utilization of the various devices examined were not the focus of this study. Nevertheless, Knitter et al*.* performed an extensive cost analysis of L- *vs.* R-MILS of patients who experienced major hepatectomies for benign and malign liver tumors in Germany [49]. The authors concluded that median daily and total costs were comparable between robotic and laparoscopic surgery (16648 € *vs.* 14578 €). It could be noted that about 50% of R-MILS costs were caused by intraoperative costs (7,592 €). Therefore, the systematic review and network meta-analysis by Koh et al*.* evaluated 45 studies analyzing costs-morbidity, costs-mortality, and costs-efficacy of open, laparoscopic and robotic liver surgery [50]. In summary, the analysis indicates that L-MILS is a cost-effective option for hepatectomy, associated with improved postoperative morbidity and mortality, as well as shorter hospital stays.

Furthermore, the limited availability of robotic platforms—often restricted to less than 24 h a day and 7 days a week in most centers—may represent a potential bias influencing both the indications for and the actual utilization of robotic systems. This constraint could inherently limit the applicability of findings to clinical settings with unrestricted access, thereby impacting the generalizability of the results.

Our survey indicates a growing preference for robotic approaches in complex liver surgeries, but laparoscopic surgery remains still widely adopted. Interestingly, CUSA use was more frequent in major hepatic resections, yet there was no statistically significant difference between major and minor procedures regarding its preference. This suggests that centers rely on CUSA irrespective of case complexity, valuing its precision and efficacy in liver transection, especially expressed by HPB dedicated surgeons. On the other hand, very well-trained robotic surgeons may face liver resections only using advanced bipolar scissors. It is clear that there is still a clear gap in robotic instrumentation, necessitating hybrid techniques. These findings reflect a blend of traditional and robotic approaches, showcasing the incomplete transition to fully robotic liver resection techniques.

Other aspects that should be underline are the challenges of transecting liver tissue, where CUSA’s precision is favored over other devices.

Furthermore, we highlight a strong reliance on topical agents for achieving hemostasis in liver surgery. These findings reinforce the need for better robotic instrumentation and wider adoption of minimally invasive techniques to optimize liver surgery outcomes and diffusion.

### Limitations

The outcomes of this survey should be interpreted in the light of several limitations. First, this survey undoubtedly contains a degree of selection bias toward centers performing MILS since all participants performed MILS. Second, the total number of surgeons who received this survey was a partial percentage of all, and any high-volume centers didn’t participate. Furthermore, current study cut-off value to categorize a center as lower- or higher-volume center was > 50 liver resections per year based on descriptive data without asking responding surgeons whether they would consider their liver surgery practice low- or high-volume. This cut-off may be controversial since there is limited previously published data regarding the optimal cut-off to define a high-volume center.

Furthermore, our study does not explore the costs associated with the devices used in hepatic surgery: The cost analysis cannot be effectively conducted due to the heterogeneity of the healthcare systems referenced in this study. This challenge arises both from the international scope of the survey and the federal nature of the Italian healthcare system, where procedural costs vary significantly across different regions.

Another key factor influencing costs is the volume of procedures performed, and the various commercial agreements established with medical device companies. While it is evident that the use of laparoscopic devices in robotic surgery increases overall costs, fully laparoscopic procedures remain more economically favorable compared to robotic approaches. However, this cost paradigm may soon shift with the introduction of new robotic devices and surgical robotic platforms, potentially altering the economic landscape of minimally invasive liver surgery.

Another limitation is the lack of outcomes analysis in reconstructive hepatic surgery employing robotic or laparoscopic instruments, which will be addressed in a future study.

## Conclusion

Minimally invasive management of liver parenchymal transection represents one of the main aspects of this surgery. MILS seems well implemented with laparoscopy still being the most common approach. CUSA devices represent the most effective device in major hepatectomies, while ultrasonic shears and electrosurgical pencil and advanced bipolar devices in minor resections. Despite those evidence, there are still very well-trained robotic surgeons that approach major hepatectomies only using advanced bipolar and robotic devices.

R-MILS overcome several limitations of laparoscopic instruments thanks to 3D vision, a 360° EndoWrist degree of movements, and effectiveness in vascular and biliary reconstructions. Nevertheless, R-MILS allows good results during liver transection with the clamp-crush technique or hybrid approaches.

Although robotic costs should be further assessed, the development of dedicated robotic TDs will lead to an increase in the adoption of R-MILS.

## Supplementary Information

Below is the link to the electronic supplementary material.

Supplementary file1 (DOCX 48 KB)

Supplementary file2 (DOCX 500 KB)

## Data Availability

The raw data supporting the conclusions of this article will be made available by the authors, with undue reservation.
