# Supplementary material for: Devices for minimally invasive liver parenchyma transection: the SICE (Italian Society of Endoscopic Surgery) Italian and International survey
Source: Surg Endosc. 2025 Jun 16;39(8):4822–38. doi: 10.1007/s00464-025-11769-3 (PMC12287225; doi:10.1007/s00464-025-11769-3)
Supplement: Supplementary file 1 — Supplementary file1 (DOCX 48 KB) [file 464_2025_11769_MOESM1_ESM.docx]

**Appendix 1. Devices for Minimally Invasive Liver Parenchyma Transection**

**The SICE (Italian Society of Endoscopic Surgery) International survey**

#### Name, Surname (Principal Investigator)

#### Other collaborators (max 2)

#### Institution

1. E-mail
2. ORCID (Optional)
3. What is your age?
4. In which country do you work? (Menù a tendina)
5. In what type of medical centre are you working?
   1. Private hospital (central hospital)
   2. Public hospital (central hospital)
   3. Private hospital (peripherical hospital)
   4. Public hospital (peripherical hospital)
6. What best describes the scope of your current clinical practice?
   1. General surgery
   2. Surgical oncology
   3. Gastrointestinal surgery
   4. HPB surgery
   5. Liver surgery
   6. Other:
7. Do you have a robot device in your current center?
   1. Yes
   2. No
8. If yes, do you use the robot for all general surgery procedures?
   1. Yes
   2. No
9. Per year, how many liver resections are performed yearly in your center?
   1. <20 resections/year (Low volume)
   2. 20-50 resections/year (Mid volume)
   3. ≥50 resections/year (High volume)
10. Is minimally invasive liver surgery (laparoscopic and/or robotic surgery) performed in your center?
    1. Yes
    2. No
11. If yes, which type of minimally invasive liver resections are performed in your center?
    1. Laparoscopic
    2. Robot-assisted
    3. Both
12. Per year, how many **laparoscopic liver** resections are performed in total in your center?
    1. <20%
    2. 21-30%
    3. 31-50%
    4. >50%
13. If robot console is available in your center: per year, how many **robotic liver resections** are performed in total in your center?
    1. <20%
    2. 21-30%
    3. 31-50%
    4. >50%
14. What percentage of all **MINOR** laparoscopic liver resections is performed in your center?
    1. 0-25%
    2. 26-50%
    3. 51-75%
    4. 76-100%
15. What percentage of all **MINOR** robotic liver resections is performed in your center?
    1. 0-25%
    2. 26-50%
    3. 51-75%
    4. 76-100%
16. What percentage of all **MAJOR (≥3 liver segments)** laparoscopic liver resections is performed in your center?
    1. 0-25%
    2. 26-50%
    3. 51-75%
    4. 76-100%
17. What percentage of all **MAJOR (≥3 liver segments)** robotic liver resections is performed in your center?
    1. 0-25%
    2. 26-50%
    3. 51-75%
    4. 76-100%
18. In case of challenging procedures (IWATE >6) or major hepatectomies (≥3 liver segments), which kind of approach do you perform?
    1. Laparoscopic
    2. Robotic
    3. Both
19. Which system is primarily used for robotic liver resections?
    1. Yes, with da Vinci Xi, Intuitive Surgical
    2. Yes, with da Vinci X, Intuitive Surgical
    3. Yes, with da Vinci Si, Intuitive Surgical
    4. Yes, Hugo RAS, Medtronic
    5. Yes, other (free text)
    6. Not available at my center
20. How long time have you been performing robotic liver resections?
    1. Since less than 1 year
    2. Between one to 3 years
    3. Between three to 5 years
    4. Since more than 5 years
21. Do you choose dedicated devices according to segment resection?
    1. Yes
    2. No
22. What is your mainly used DEVICE for **Laparoscopic MINOR** parenchymal transections? (percentage of use)
    1. Laparoscopic Monopolar (Hook/Scissor)
    2. Bipolar energy devices
    3. Monopolar and Bipolar cautery
    4. Ultrasonic shears/scalpel energy devices: Lotus Torsional® (BOWA Medical, UK), Harmonic® (Ethicon Endo-Surgery, USA), Sonicision™ (Medtronic, USA), HARMONIC ACE® + 7 Shears (Ethicon Endo-Surgery, USA), Ultracision® (Covidien, USA) SonoSurg® (Conmed, SS)
    5. Advanced bipolar devices: LigaSure™ (Medtronic, USA), ENSEAL (Ethicon Endo-Surgery, USA)
    6. Advanced bipolar and ultrasonic systems: THUNDERBEAT® (Olympus, Japan)
    7. Stapler (*eg.* Endostapler) & Reinforcement
    8. Monopolar or Bipolar Radiofrequency needle: Habib^TM^ 4X (LH4X, Rita, USA), Aquamantys™ (Medtronic, Minneapolis, MN, USA)
    9. Saline-linked radiofrequency dissector (TissueLink medical's DS3.5, Dover, NH)
    10. Cavitronic Ultrasonic Surgical Aspirator (CUSA EXcel®)
    11. Water-jet (Helix Hydro-Jet, Erbe, Tubingen, Germany)
    12. Pre-coagulators (Radiotherapy-assisted devices)
    13. Argon beam coagulation
    14. Others (text to specify)
23. What is your mainly used DEVICE for Laparoscopic MAJOR parenchymal transections? (percentage of use)
    1. Laparoscopic Monopolar (Hook/Scissor)
    2. Bipolar energy devices
    3. Monopolar and Bipolar cautery
    4. Ultrasonic shears/scalpel energy devices: Lotus Torsional® (BOWA Medical, UK), Harmonic® (Ethicon Endo-Surgery, USA), Sonicision™ (Medtronic, USA), HARMONIC ACE® + 7 Shears (Ethicon Endo-Surgery, USA), Ultracision® (Covidien, USA) SonoSurg® (Conmed, SS)
    5. Advanced bipolar devices: LigaSure™ (Medtronic, USA), ENSEAL (Ethicon Endo-Surgery, USA)
    6. Advanced bipolar and ultrasonic systems: THUNDERBEAT® (Olympus, Japan)
    7. Stapler (*eg.* Endostapler) & Reinforcement
    8. Monopolar or Bipolar Radiofrequency needle: Habib^TM^ 4X (LH4X, Rita, USA), Aquamantys™ (Medtronic, Minneapolis, MN, USA)
    9. Saline-linked radiofrequency dissector (TissueLink medical's DS3.5, Dover, NH)
    10. Cavitronic Ultrasonic Surgical Aspirator (CUSA EXcel®)
    11. Water-jet (Helix Hydro-Jet, Erbe, Tubingen, Germany)
    12. Pre-coagulators (Radiotherapy-assisted devices)
    13. Argon beam coagulation
    14. Others (text to specify)
24. What is your mainly used dedicated DEVICE for **Robotic** **MINOR** parenchymal transections?
    1. Robotic Maryland bipolar
    2. Robotic Harmonic scalpel
    3. Robotic Vesselsealer (Da Vinci SynchroSeal)
    4. Robotic vascular staplers
    5. Robotic scissors
    6. Robotic Hook
    7. Robotic Argon beam device
    8. Robotic Hem-o-lok
    9. Combining other laparoscopic devices (text)
25. What is your mainly used TECHNIQUE during **Robotic MAJOR** parenchymal transections?
    1. Robotic Maryland bipolar
    2. Robotic Harmonic scalpel
    3. Robotic Vesselsealer (Da Vinci SynchroSeal)
    4. Robotic vascular staplers
    5. Robotic scissors
    6. Robotic Hook
    7. Robotic Argon beam device
    8. Robotic Hem-o-lok
    9. Combining other laparoscopic devices
26. Technique of Robotic liver dissection (minor/major)
    1. Clamp-crushing
    2. Cavitation
    3. Layer-by-layer Transection (harmonic, scissors, hook, etc)
    4. Stapler transection
    5. Snip-electrocoagulation
    6. Radiofrequency energy (Habib)
    7. Combined/hibrid technique
27. What is your mainly used technic for open parenchymal transection?
    1. Ultrasonic (*e.g.* CUSA)
    2. Radiofrequency (*e.g*. Salient Dissecting Sealer, radiofrequency probe)
    3. Ultrasonic + radiofrequency
    4. Bipolar (*e.g*. Ligasure)
    5. Stapler (*e.g.* Endo surgical stapler)
    6. Radiofrequency needle
    7. Other
28. Which type of hemostatic technique do you prefer?
    1. Knot-type ligation, stitches for vessel management (mainly)
    2. Titanium clips for vessel management (mainly)
    3. Polymer Ligation Clip System (Hem-o-lock™ clips, Weck, USA) for vessel management (mainly)
    4. Others (text to specify)
29. Which type of Intra-Operative Ultra-Sound (IOUS) do you use in robotic surgery?
    1. Dedicated robotic probes
    2. Laparoscopic probes
30. Are you used to prepare an inflow vascular control before liver transection (MINOR/MAJOR)?
    1. Yes
    2. No
    3. In selected cases
31. Do you perform the Pringle maneuver or selected vascular control during liver resection?
    1. Yes
    2. No
32. Are you used to modify the liver resection device according to anatomical or non-anatomical resection (minor/major)?
    1. Yes (🡪 text to specify)
    2. No
33. Do you use a “one-surgeon or two-surgeon” technique during parenchyma transection?
    1. One-surgeon
    2. Two-surgeon
34. Are you used to modify the liver resection device according to liver parenchyma?
    1. Yes (🡪 text to specify)
    2. No
35. Do you use different techniques if the liver is cirrhotic?
    1. Yes
    2. No
36. If yes, what is your mainly used technic for cirrhotic parenchymal transection?
    1. Ultrasonic (*e.g.* CUSA)
    2. Radiofrequency (*e.g*. Salient Dissecting Sealer, radiofrequency probe)
    3. Ultrasonic + radiofrequency
    4. Bipolar (*e.g*. Ligasure)
    5. Stapler (*e.g.* Endo surgical stapler)
    6. Radiofrequency needle
    7. Monopolar hook
    8. Monopolar scissors
    9. Extended grasper (*e.g.* double-fenestrated grasper)
    10. Vessel sealer
    11. Other
37. Which option do you consider more important in MILS a 3D or 4K view?
    1. 3D
    2. 4K
    3. Both of them
38. Are you using any of these?
    1. 3D
    2. 4K
    3. Both of them
39. Are you satisfied with the available robotic instrumentation for liver resection?
    1. Yes
    2. No
40. If not, what would you like to have in addition? Please describe
    1. Text to specify
41. Do you use any classification to decide which device to use?
    1. Yes
    2. No
42. If yes, what type of classification do you use?
    1. Text to specify
43. Are you used to apply any topical sealants on the liver surface after hepatectomy?
    1. Collagen based sealants (eg. Tissucol)
    2. Fibrinogen based sealant (eg. Tachosil)
    3. Others (text to specify)
44. Are you used to use Indocyanine Green (ICG) imaging for liver resection?
    1. Yes
    2. No
    3. In selected cases (text to specify)

**References**

- - - 1. Yoh T, Cauchy F, Soubrane O. Techniques for laparoscopic liver parenchymal transection. Hepatobiliary Surg Nutr. 2019 Dec;8(6):572-581. doi: 10.21037/hbsn.2019.04.16.
      2. Somasundar P, Boutros C, Helton WS, Espat NJ. Evaluation of a bipolar radiofrequency device for laparoscopic hepatic resection: technique and clinical experience in 18 patients. HPB (Oxford). 2009 Mar;11(2):145-9. doi: 10.1111/j.1477-2574.2009.00026.x.
      3. Houben, P., Khajeh, E., Hinz, U. *et al.* SEALIVE: the use of technical vessel-sealing devices for recipient hepatectomy in liver transplantation: study protocol for a randomized controlled trial. *Trials* **19**, 380 (2018). <https://doi.org/10.1186/s13063-018-2778-1>
      4. Toyama Y, Yoshida S, Saito R, Iwase R, Haruki K, Okui N, Shimada J, Kitamura H, Matsumoto M, Yanaga K. Efficacy of a half-grip technique using a fine tip LigaSure™, Dolphin Tip Sealer/Divider, on liver dissection in swine model. BMC Res Notes. 2015 Aug 20;8:362. doi: 10.1186/s13104-015-1316-4.
      5. Pai M, Spalding D, Jiao L, Habib N. Use of bipolar radiofrequency in parenchymal transection of the liver, pancreas and kidney. Dig Surg. 2012;29(1):43-7. doi: 10.1159/000335732. Epub 2012 Mar 15.
      6. Aloia TA, Zorzi D, Abdalla EK, Vauthey JN. Two-surgeon technique for hepatic parenchymal transection of the noncirrhotic liver using saline-linked cautery and ultrasonic dissection. Ann Surg. 2005 Aug;242(2):172-7. doi: 10.1097/01.sla.0000171300.62318.f4.
      7. Lesurtel M, Selzner M, Petrowsky H, McCormack L, Clavien PA. How should transection of the liver be performed?: a prospective randomized study in 100 consecutive patients: comparing four different transection strategies. Ann Surg. 2005 Dec;242(6):814-22, discussion 822-3. doi: 10.1097/01.sla.0000189121.35617.d7.
      8. Xiao L, Wang Z, Zhou L. Snip-electrocoagulation technique versus clamp-crashing technique for parenchyma transection in liver resection: a pilot study. Ann Transl Med. 2020 Jun;8(12):744. doi: 10.21037/atm-20-3019.
      9. Jayant K, Sodergren MH, Reccia I, Kusano T, Zacharoulis D, Spalding D, Pai M, Jiao LR, Huang KW. A Systematic Review and Meta-Analysis Comparing Liver Resection with the Rf-Based Device Habib™-4X with the Clamp-Crush Technique. Cancers (Basel). 2018 Nov 8;10(11):428. doi: 10.3390/cancers10110428.
      10. Fujikawa T, Kajiwara M. Modified Two-Surgeon Technique for Laparoscopic Liver Resection. Cureus. 2022 Mar 27;14(3):e23528. doi: 10.7759/cureus.23528.
      11. Fujikawa T, Uemoto Y, Matsuoka T, Kajiwara M. Novel Liver Parenchymal Transection Technique Using Saline-linked Monopolar Cautery Scissors (SLiC-Scissors) in Robotic Liver Resection. Cureus. 2022 Aug 17;14(8):e28118. doi: 10.7759/cureus.28118.
      12. Kajiwara M, Fujikawa T, Naito S, Sasaki T, Nakashima R, Hasegawa S. Non-Stick Liver Parenchymal Transection With Saline-Linked Bipolar Clamp-Crush Technique in Robotic Liver Resection. Cureus. 2023 Mar 20;15(3):e36401. doi: 10.7759/cureus.36401.
      13. Uemoto Y, Fujikawa T, Matsuoka T. Laparoscopic Liver Resection Utilizing a Water Jet Scalpel for Patients With Liver Fibrosis. Cureus. 2023 Sep 14;15(9):e45212. doi: 10.7759/cureus.45212.
      14. Qu Z, Wu KJ, Feng JW, Shi DS, Chen YX, Sun DL, Duan YF, Chen J, He XZ. Treatment of hepatic venous system hemorrhage and carbon dioxide gas embolization during laparoscopic hepatectomy *via* hepatic vein approach. Front Oncol. 2023 Jan 5;12:1060823. doi: 10.3389/fonc.2022.1060823.
      15. Dang KT, Naka S, Yamada A, Tani T. Feasibility of Microwave-Based Scissors and Tweezers in Partial Hepatectomy: An Initial Assessment on Canine Model. Front Surg. 2021 Jun 17;8:661064. doi: 10.3389/fsurg.2021.661064.
      16. S Hammond J, Muirhead W, Zaitoun AM, Cameron IC, Lobo DN. Comparison of liver parenchymal ablation and tissue necrosis in a cadaveric bovine model using the Harmonic Scalpel, the LigaSure, the Cavitron Ultrasonic Surgical Aspirator and the Aquamantys devices. HPB (Oxford). 2012 Dec;14(12):828-32. doi: 10.1111/j.1477-2574.2012.00547.x. Epub 2012 Aug 26.
      17. Itano O, Ikoma N, Takei H, Oshima G, Kitagawa Y. The superficial precoagulation, sealing, and transection method: a "bloodless" and "ecofriendly" laparoscopic liver transection technique. Surg Laparosc Endosc Percutan Tech. 2015 Feb;25(1):e33-e36. doi: 10.1097/SLE.0000000000000051.
      18. Sotiropoulos GC, Stamopoulos P, Charalampoudis P, Molmenti EP, Voutsarakis A, Kouraklis G. Totally laparoscopic left hepatectomy using the Torsional Ultrasonic Scalpel. World J Gastroenterol. 2013 Sep 21;19(35):5929-32. doi: 10.3748/wjg.v19.i35.5929.
      19. Kajiwara M, Fujikawa T, Hasegawa S. Tissue pad degradation of ultrasonic device may enhance thermal injury and impair its sealing performance in liver surgery. World J Hepatol. 2022 Jul 27;14(7):1357-1364. doi: 10.4254/wjh.v14.i7.1357. PMID: 36158911; PMCID: PMC9376783.
