# Supplementary material for: Devices for minimally invasive liver parenchyma transection: the SICE (Italian Society of Endoscopic Surgery) Italian and International survey
Source: Surg Endosc. 2025 Jun 16;39(8):4822–38. doi: 10.1007/s00464-025-11769-3 (PMC12287225; doi:10.1007/s00464-025-11769-3)
Supplement: Supplementary file 2 — Supplementary file2 (DOCX 500 KB) [file 464_2025_11769_MOESM2_ESM.docx]

| **First name and Last name** | **Email** | **ORCID** | **Institution** |
| --- | --- | --- | --- |
| Davide Giovanni Grego | DGGrego@asst-rhodense.it |  | HPB, Emergency Surgery & Liver Surgery at ASST Rhodense - Milan, Italy |
| Roberto Lauro | rlauro62@gmail.com | 0000-0001-7344-9472 | HPB, Emergency Surgery & Liver Surgery at ASST Rhodense - Milan, Italy |
| Nicolas Pontarolo | NPontarolo@asst-rhodense.it |  | HPB, Emergency Surgery & Liver Surgery at ASST Rhodense - Milan, Italy |

| **First name and Last name** | **Email** | **ORCID** | **Institution** |
| --- | --- | --- | --- |
| Jacopo Andreuccetti | jacopo.andreuccetti@gmail.com | 0000-0003-0474-9775 | General Surgery 2, ASST Spedali Civili of Brescia, Italy |
| Rossella D’Alessio | dalessio.rossella87@gmail.com | 0000-0001-5614-7616 | General Surgery 2, ASST Spedali Civili of Brescia, Italy |
| Giusto Pignata | GiustoPignata@gmail.com | 0000-0002-2381-1055 | General Surgery 2, ASST Spedali Civili of Brescia, Italy |

| **First name and Last name** | **Email** | **ORCID** | **Institution** |
| --- | --- | --- | --- |
| Giuseppe Calaciura | giuseppe.calaciura96@gmail.com | 0009-0009-2327-063X | Hepatobiliary and Pancreatic Surgery Unit ARNAS Garibaldi Hospital, Catania, Italy |
| Nicola Cinardi | nicocinardi@hotmail.com | 0009-0006-0856-9658 | Hepatobiliary and Pancreatic Surgery Unit ARNAS Garibaldi Hospital, Catania, Italy |
| Riccardo Schillaci | riccardo.schillaci1988@gmail.com | 0000-0003-0394-0543 | Hepatobiliary and Pancreatic Surgery Unit ARNAS Garibaldi Hospital, Catania, Italy |

| **First name and Last name** | **Email** | **ORCID** | **Institution** |
| --- | --- | --- | --- |
| Alessandro Mazzotta | alex.mazzotta@gmail.com |  | Department of Surgery, M.G. General Vannini Hospital, Istituto Figlie Di San Camillo, Rome, Italy |

| **First name and Last name** | **Email** | **ORCID** | **Institution** |
| --- | --- | --- | --- |
| Roberta Angelico | roberta.angelico@uniroma2.it | 0000-0002-3439-7750 | Hepatobiliary Surgery and Transplant Unit, Department of Surgical Sciences, University of Rome Tor Vergata, 00133 Rome, Italy |
| Luigi Eduardo Conte | conteluigieduardo@gmail.com | 0000-0002-1280-8029 | Hepatobiliary Surgery and Transplant Unit, Department of Surgical Sciences, University of Rome Tor Vergata, 00133 Rome, Italy |
| Tommaso Maria Manzia | manzia@med.uniroma2.it | 0000-0002-4636-3478 | Hepatobiliary Surgery and Transplant Unit, Department of Surgical Sciences, University of Rome Tor Vergata, 00133 Rome, Italy |

| **First name and Last name** | **Email** | **ORCID** | **Institution** |
| --- | --- | --- | --- |
| Marco Anania | ananiamarco@gmail.com | 0000-0002-4202-6968 | Department of Medicine, Surgery and Pharmacy, University of Sassari, Sassari, Italy |
| Nicola Mancini | nic.mancini97@gmail.com |  | Department of Medicine, Surgery and Pharmacy, University of Sassari, Sassari, Italy |
| Marco Pazzona | makong84@gmail.com |  | Department of Medicine, Surgery and Pharmacy, University of Sassari, Sassari, Italy |

| **First name and Last name** | **Email** | **ORCID** | **Institution** |
| --- | --- | --- | --- |
| Giuseppe Frazzetta | giuseppe.frazzetta@arnascivico.it | 0000-0003-2505-4972 | Oncologic Surgery Unit, ARNAS Civico Hospital, Palermo 90100, Italy |
| Antonio Picciurro | antonino.picciurro@arnascivico.it |  | Oncologic Surgery Unit, ARNAS Civico Hospital, Palermo 90100, Italy |

| **First name and Last name** | **Email** | **ORCID** | **Institution** |
| --- | --- | --- | --- |
| Annarita Libia | libiamd@me.com |  | HPB and General Surgery Unit, Vito Fazzi Hospital, Lecce, Italy |
| Marcello G. Spampinato | marcello.spampinato@gmail.com | 0000-0003-4036-6819 | HPB and General Surgery Unit, Vito Fazzi Hospital, Lecce, Italy |

| **First name and Last name** | **Email** | **ORCID** | **Institution** |
| --- | --- | --- | --- |
| Cosimo Saviello | saviello.cosimo@gmail.com | 0000-0002-9379-4334 | Department of Medicine and Surgery, University of Salerno, Italy |

| **First name and Last name** | **Email** | **ORCID** | **Institution** |
| --- | --- | --- | --- |
| Giovanni Spiezio | giovanni.spiezio90@gmail.com | 0000-0002-3164-690X | General Surgery Unit, San Carlo Borromeo Hospital, Milano, Italy |

| **First name and Last name** | **Email** | **ORCID** | **Institution** |
| --- | --- | --- | --- |
| Emanuele Pontecorvi | emanuelepontecorvi@virgilio.it | 0000-0002-8711-9287 | Department of General Surgery, SS Annunziata Hospital, 67039, Sulmona, L'Aquila, Italy |
| Vania Silvestri | vaniasilvestri83@gmail.com | 0000-0002-2754-2382 | Department of General Surgery, SS Annunziata Hospital, 67039, Sulmona, L'Aquila, Italy |

| **First name and Last name** | **Email** | **ORCID** | **Institution** |
| --- | --- | --- | --- |
| Teresa Perra | teresaperra1992@gmail.com | 0000-0001-7032-1289 | Unit of General Surgery 2, Department of Medical, Surgical and Experimental Sciences, University of Sassari, 07100, Sassari, Italy |
| Alberto Porcu | alberto.porcu.1@gmail.com | 0000-0001-6307-8938 | Unit of General Surgery 2, Department of Medical, Surgical and Experimental Sciences, University of Sassari, 07100, Sassari, Italy |

| **First name and Last name** | **Email** | **ORCID** | **Institution** |
| --- | --- | --- | --- |
| Alberto Oldani | alberto.oldani@libero.it |  | Surgeon of General and Oncologic Surgery Department, Centre of Advanced Laparoscopic Surgery, Centre of Bariatric Surgery, San Marco Hospital GSD, Zingonia, Italy |
| Stefano Olmi | stefano.olmi@gmail.com | 0000-0002-8307-3402 | Surgeon of General and Oncologic Surgery Department, Centre of Advanced Laparoscopic Surgery, Centre of Bariatric Surgery, San Marco Hospital GSD, Zingonia, Italy |

| **First name and Last name** | **Email** | **ORCID** | **Institution** |
| --- | --- | --- | --- |
| Alessandra Brescacin | brescacin.alessandra@gmail.com | 0000-0002-7030-8233 | Humanitas University, Milan, Italy |

| **First name and Last name** | **Email** | **ORCID** | **Institution** |
| --- | --- | --- | --- |
| Giampaolo Formisano | giampaolo.formisano@unimi.it |  | Division of General and Robotic Surgery, Department of Health Sciences, University of Milan, 20142 Milano, Italy |

| **First name and Last name** | **Email** | **ORCID** | **Institution** |
| --- | --- | --- | --- |
| Matteo Barabino | mbarabino2@gmail.com | 0000-0001-9525-9063 | Department of Health Sciences (DISS), University of Milan, San Paolo Hospital, Milan 20142, Lombardy, Italy |
| Gaetano Piccolo | gaetano.piccolo@asst-santipaolocarlo.it | 0000-0002-4942-7705 | Department of Health Sciences (DISS), University of Milan, San Paolo Hospital, Milan 20142, Lombardy, Italy |

| **First name and Last name** | **Email** | **ORCID** | **Institution** |
| --- | --- | --- | --- |
| Andrea Barberis | andrea.barberis@galliera.it | 0000-0003-0472-0120 | General and Hepatobiliary Surgery, E.O. Ospedale Galliera, 16128 Genoa, Italy |

| **First name and Last name** | **Email** | **ORCID** | **Institution** |
| --- | --- | --- | --- |
| Giovanni Tebala | gtebala@gmail.com | 0000-0001-7152-4096 | Department of Digestive and Emergency Surgery, S.Maria Hospital Trust, Terni, Italy |

| **First name and Last name** | **Email** | **ORCID** | **Institution** |
| --- | --- | --- | --- |
| Andrea Coratti | corattian@gmail.com |  | Misericordia Hospital. Department of General and Emergency surgery. School of robotic surgery. Grosseto |
| Giuseppe Giuliani | Giu.giuliani86@gmail.com | 0000-0001-9232-398X | Misericordia Hospital. Department of General and Emergency surgery. School of robotic surgery. Grosseto |
| Francesco Guerra | fra.guerra.mail@gmail.com | 0000-0003-2891-4659 | Misericordia Hospital. Department of General and Emergency surgery. School of robotic surgery. Grosseto |

| **First name and Last name** | **Email** | **ORCID** | **Institution** |
| --- | --- | --- | --- |
| Andrea Morini | andrea.morini@ausl.re.it | 0000-0001-9140-0488 | Reggio Emilia Local Agency - IRCCS Advanced Technologies and Care Models in Oncology, Surgical Oncology Unit, Reggio Emilia, Italy |
| Massimiliano Fabozzi | massimiliano.fabozzi@ausl.re.it |  | Reggio Emilia Local Agency - IRCCS Advanced Technologies and Care Models in Oncology, Surgical Oncology Unit, Reggio Emilia, Italy |
| Maurizio Zizzo | maurizio.zizzo@ausl.re.it |  | Reggio Emilia Local Agency - IRCCS Advanced Technologies and Care Models in Oncology, Surgical Oncology Unit, Reggio Emilia, Italy |

| **First name and Last name** | **Email** | **ORCID** | **Institution** |
| --- | --- | --- | --- |
| Bruno Nardo | bruno.nardo.editor@gmail.com | 0000-0001-7263-860X | Department of Surgery, Annunziata Hospital of Cosenza, Cosenza, Italy |
| Daniele Paglione | danielepagli1@gmail.com | 0000-0003-3922-6552 | Department of Surgery, Annunziata Hospital of Cosenza, Cosenza, Italy |
| Francesco Pata | francesco.pata@gmail.com | 0000-0003-2634-1199 | Department of Surgery, Annunziata Hospital of Cosenza, Cosenza, Italy |

| **First name and Last name** | **Email** | **ORCID** | **Institution** |
| --- | --- | --- | --- |
| Alexander Julianov | a_julianov@yahoo.com | 0000-0003-4874-9611 | Trakia Hospital |
| Azize Saroglu | azize_saroglu@hotmail.com | 0000-0001-7675-7657 | Trakia Hospital |

| **First name and Last name** | **Email** | **ORCID** | **Institution** |
| --- | --- | --- | --- |
| Mattia Garancini | Mattia.garancini@irccs-sangerardo.it |  | School of Medicine and Surgery, University of Milano-Bicocca, San Gerardo Hospital, Monza, Italy |
| Fabrizio Romano | fabrizio.romano@unimib.it | 0000-0001-5341-0706 | School of Medicine and Surgery, University of Milano-Bicocca, San Gerardo Hospital, Monza, Italy |
| Mauro Alessandro Scotti | Mauro Alessandro.scotti@irccs-sangerardo.it |  | School of Medicine and Surgery, University of Milano-Bicocca, San Gerardo Hospital, Monza, Italy |

| **First name and Last name** | **Email** | **ORCID** | **Institution** |
| --- | --- | --- | --- |
| Andrea Quazzico | andreaquazzico87@gmail.com | 0009-0001-8708-6681 | Department of Medical and Surgical Sciences, University of Foggia, Foggia, Italy |

| **First name and Last name** | **Email** | **ORCID** | **Institution** |
| --- | --- | --- | --- |
| Stefano D’Ugo | dugo.stefano@gmail.com | 0000-0002-7073-0565 | HPB and General Surgery Unit, Vito Fazzi Hospital, Lecce, Italy |
| Annarita Libia | libiamd@me.com |  | HPB and General Surgery Unit, Vito Fazzi Hospital, Lecce, Italy |
| Marcello Spampinato | marcello.spampinato@gmail.com |  | HPB and General Surgery Unit, Vito Fazzi Hospital, Lecce, Italy |

| **First name and Last name** | **Email** | **ORCID** | **Institution** |
| --- | --- | --- | --- |
| Fabio Giannone | fabio.giannone@ospedale.al.it | 0000-0001-5065-6962 | SCDU General Surgery, Surgical Oncology, Robotic and HBP Surgery, AOUAL SS. Antonio e Biagio e Cesare Arrigo, University of Eastern Piedmont, Alessandria, Italy |
| Fabrizio Panaro | fabrizio.panaro@ospedale.al.it |  | SCDU General Surgery, Surgical Oncology, Robotic and HBP Surgery, AOUAL SS. Antonio e Biagio e Cesare Arrigo, University of Eastern Piedmont, Alessandria, Italy |
| Federico Sangiuolo | federico.sangiuolo@ospedale.al.it |  | SCDU General Surgery, Surgical Oncology, Robotic and HBP Surgery, AOUAL SS. Antonio e Biagio e Cesare Arrigo, University of Eastern Piedmont, Alessandria, Italy |

| **First name and Last name** | **Email** | **ORCID** | **Institution** |
| --- | --- | --- | --- |
| Giulia Lauteri | giulia.lauteri@uniroma1.it | 0009-0003-9349-335X | General Surgery San Carlo di Nancy Hospital, Rome, Italy |
| Federico Maggi | maggi.fede6@gmail.com | 0009-0002-4103-3979 | General Surgery San Carlo di Nancy Hospital, Rome, Italy |
| Luigi Masoni | surgerymas@gmail.com | 0000-0001-9866-4224 | General Surgery San Carlo di Nancy Hospital, Rome, Italy |

| **First name and Last name** | **Email** | **ORCID** | **Institution** |
| --- | --- | --- | --- |
| Marcella Arru | marcella_arru@asst-bgovest.it | 0000-0002-3113-2220 | Surgery Unit, ASST Bergamo Ovest, Treviglio, BG, Italy |
| Matteo Viti | matteo_viti@asst-bgovest.it |  | Surgery Unit, ASST Bergamo Ovest, Treviglio, BG, Italy |

| **First name and Last name** | **Email** | **ORCID** | **Institution** |
| --- | --- | --- | --- |
| Belkacem Acidi | gacem05@hotmail.com |  | CHB - Centre Hépato-Biliaire, Hôpital Paul Brousse, France |

| **First name and Last name** | **Email** | **ORCID** | **Institution** |
| --- | --- | --- | --- |
| Sang Thanh Nguyen | drthanhsangnguyenbvtv@gmail.com |  | Department of General Surgery, Trung Vuong hospital, Ho Chi Minh city, Viet Nam |

| **First name and Last name** | **Email** | **ORCID** | **Institution** |
| --- | --- | --- | --- |
| Michele Ammendola | michele.ammendola@unicz.it |  | Health of Science Department, "Magna Graecia" University, Digestive Surgery Unit, "R.Dulbecco" University Hospital, Catanzaro, Italy |
| Francesca Vescio | vescio.francesca@gmail.com |  | Health of Science Department, "Magna Graecia" University, Digestive Surgery Unit, "R.Dulbecco" University Hospital, Catanzaro, Italy |

| **First name and Last name** | **Email** | **ORCID** | **Institution** |
| --- | --- | --- | --- |
| Tommaso Fontana | tommasofontana2@virgilio.it | 0000-0002-1511-855X | General Surgery Unit, P.O. "Vittorio Emanuele" Gela, Italy |

| **First name and Last name** | **Email** | **ORCID** | **Institution** |
| --- | --- | --- | --- |
| Flavio Milana | milana.flavio@gmail.com | 0000-0003-3570-9573 | Department of Surgery - Division of Hepatobiliary & General Surgery Humanitas University, Rozzano - Milan, Italy. |
| Fabio Procopio | fabio.procopio@hunimed.eu |  | Department of Surgery - Division of Hepatobiliary & General Surgery Humanitas University, Rozzano - Milan, Italy. |
| Guido Torzilli | guido.torzilli@hunimed.eu |  | Department of Surgery - Division of Hepatobiliary & General Surgery Humanitas University, Rozzano - Milan, Italy. |

| **First name and Last name** | **Email** | **ORCID** | **Institution** |
| --- | --- | --- | --- |
| Giulio Argenio | giulio.argenio@hotmail.it | 0000-0001-9187-9003 | General and Oncological Surgery Unit, AORN San Pio, Benevento, Italy |

| **First name and Last name** | **Email** | **ORCID** | **Institution** |
| --- | --- | --- | --- |
| Giorgio Ammerata | giorgiosala4@gmail.com | 0000-0001-5485-5221 | General Surgery Renato Dulbecco University Hospital, Catanzaro |
| Giuseppe Currò | currog@unicz.it | 0000-0001-9566-1378 | General Surgery Renato Dulbecco University Hospital, Catanzaro |
| Giuseppe Sena | gspp.sena@gmail.com | 0000-0001-9793-3250 | General Surgery Renato Dulbecco University Hospital, Catanzaro |

| **First name and Last name** | **Email** | **ORCID** | **Institution** |
| --- | --- | --- | --- |
| Curci Fabio Pio | fabiopio.curci@gmail.com | 0009-0001-8631-0661 | General Surgery, "Casa Sollievo della Sofferenza Hospital", San Giovanni Rotondo, Italy |

| **First name and Last name** | **Email** | **ORCID** | **Institution** |
| --- | --- | --- | --- |
| Jose-Luis Beristain-Hernandez | jlberistain@yahoo.com | 0000-0002-7628-2177 | Instituto Mexicano del Seguro Social, Centro Médico Nacional La Raza, Hospital de Especialidades "Dr. Antonio Fraga Mouret", Servicio de Cirugía General. Ciudad de México, México |

| **First name and Last name** | **Email** | **ORCID** | **Institution** |
| --- | --- | --- | --- |
| Pietro Mezzatesta | Pmezzatesta@virgilio.it |  | Department of Surgery, La Maddalena Cancer Center, Palermo, Italy |
| Maria Cristina Saffioti | Saffioti.mariacristina@lamaddalenanet.it |  | Department of Surgery, La Maddalena Cancer Center, Palermo, Italy |

| **First name and Last name** | **Email** | **ORCID** | **Institution** |
| --- | --- | --- | --- |
| Andrea Belli | a.belli@istitutotumori.na.it |  | UOC Chirurgia Oncologica Addominale ad indirizzo Epatobiliare, Istituto Nazionale per lo studio e la cura dei Tumori, IRCCS Fondazione Pascale, Napoli |
| Francesco Izzo | f.izzo@istitutotumori.na.it | 0000-0003-3093-5408 | UOC Chirurgia Oncologica Addominale ad indirizzo Epatobiliare, Istituto Nazionale per lo studio e la cura dei Tumori, IRCCS Fondazione Pascale, Napoli |

| **First name and Last name** | **Email** | **ORCID** | **Institution** |
| --- | --- | --- | --- |
| Michele Ammendola | michele.ammendola@unicz.it |  | Health of Science Department, "Magna Graecia" University, Digestive Surgery Unit, "R.Dulbecco" University Hospital, Catanzaro, Italy |
| Francesca Vescio | vescio.francesca@gmail.com |  | Health of Science Department, "Magna Graecia" University, Digestive Surgery Unit, "R.Dulbecco" University Hospital, Catanzaro, Italy |

| **First name and Last name** | **Email** | **ORCID** | **Institution** |
| --- | --- | --- | --- |
| Graziano Ceccarelli | g.cecca2003@libero.it |  | Department of General Surgery, "Nuovo San Giovanni Battista" Hospital, Usl Umbria 2, Via M. Arcamone, 1, 06034, Foligno, PG, Italy |
| Michele De Rosa | michele.derosa@nhs.net |  | Department of General Surgery, "Nuovo San Giovanni Battista" Hospital, Usl Umbria 2, Via M. Arcamone, 1, 06034, Foligno, PG, Italy |
| Fabio Rondelli | rondellif@hotmail.com |  | Department of General Surgery, "Nuovo San Giovanni Battista" Hospital, Usl Umbria 2, Via M. Arcamone, 1, 06034, Foligno, PG, Italy |

| **First name and Last name** | **Email** | **ORCID** | **Institution** |
| --- | --- | --- | --- |
| Andrea Tufo | tufo.andrea@gmail.com | 0000-0002-4495-1241 | Surgical Unit, Ospedale del Mare, 80147 Napoli, Italy |

| **First name and Last name** | **Email** | **ORCID** | **Institution** |
| --- | --- | --- | --- |
| Raffaele Galleano | r.galleano@asl2.liguria.it | 0000-0001-9787-4908 | U.O. di Chirurgia Generale, Ospedale di Albenga, Savona |

| **First name and Last name** | **Email** | **ORCID** | **Institution** |
| --- | --- | --- | --- |
| Maurizio De Luca | nnwdel@tin.it |  | Chirurgia Generale, Ospedale Santa Maria della Misericordia, Rovigo, Italy |
| Enrico Lodo | Enrico.lodo@aulss5.veneto.it |  | Chirurgia Generale, Ospedale Santa Maria della Misericordia, Rovigo, Italy |
| Dario Parini | Dario.parini@aulss5.veneto.it |  | Chirurgia Generale, Ospedale Santa Maria della Misericordia, Rovigo, Italy |

| **First name and Last name** | **Email** | **ORCID** | **Institution** |
| --- | --- | --- | --- |
| Agostino Fernicola | agostinofernicola@yahoo.it | 0000-0002-3122-5531 | Department of Clinical Medicine and Surgery, University of Naples, "Federico II", Sergio Pansini 5, 80131, Naples, Italy |

| **First name and Last name** | **Email** | **ORCID** | **Institution** |
| --- | --- | --- | --- |
| Elio Jovine | elio.jovine@ausl.bologna.it | 0000-0003-4587-0958 | UO Chirurgia Generale e d'Urgenza IRCCS Azienda Ospedaliera Universitaria Sant'Orsola Malpighi c/o Ospedale Maggiore, Bologna, Italy |
| Laura Mastrangelo | laura.mastrangelo@ausl.bologna.it | 0000-0001-6859-0279 | UO Chirurgia Generale e d'Urgenza IRCCS Azienda Ospedaliera Universitaria Sant'Orsola Malpighi c/o Ospedale Maggiore, Bologna, Italy |

| **First name and Last name** | **Email** | **ORCID** | **Institution** |
| --- | --- | --- | --- |
| Christian Cotsoglou | christian.cotsoglou@irccs-sangerardo.it | 0000-0002-6348-0027 | Department of Gastrointestinal Surgery and Liver Transplantation, Fondazione IRCCS Istituto Nazionale Tumori, University of Milan, Milan, Italy |
| Beatrice Torre | torre.beatrice92@gmail.com | 0000-0002-7388-3241 | Department of Gastrointestinal Surgery and Liver Transplantation, Fondazione IRCCS Istituto Nazionale Tumori, University of Milan, Milan, Italy |

| **First name and Last name** | **Email** | **ORCID** | **Institution** |
| --- | --- | --- | --- |
| Mario Annecchiarico | mannecchiarico@hotmail.com |  | General and Oncological Surgery Unit, AORN San Pio, Benevento, Italy |

| **First name and Last name** | **Email** | **ORCID** | **Institution** |
| --- | --- | --- | --- |
| Luigi Bonanni | luigibonanni@yahoo.it |  | General and Transplant Surgery Department, San Salvatore Hospital, ASL 1 Abruzzo, Avezzano, Sulmona, L'Aquila, Coppito, L'Aquila, Italy |
| Davide Chiappori | chiappori.davide@gmail.com |  | General and Transplant Surgery Department, San Salvatore Hospital, ASL 1 Abruzzo, Avezzano, Sulmona, L'Aquila, Coppito, L'Aquila, Italy |
| Federico Paniccia | Federicopaniccia95@gmail.com |  | General and Transplant Surgery Department, San Salvatore Hospital, ASL 1 Abruzzo, Avezzano, Sulmona, L'Aquila, Coppito, L'Aquila, Italy |

| **First name and Last name** | **Email** | **ORCID** | **Institution** |
| --- | --- | --- | --- |
| Edoardo Saladino | edoardosaladino@hotmail.com | 0000-0003-0521-959X | Oncological Surgical Unit, Papardo Hospital, Messina, Italy |
| Elisa Bertilone | elisa.bertilone@gmail.com | 0009-0009-1658-1624 | Oncological Surgical Unit, Papardo Hospital, Messina, Italy |

| **First name and Last name** | **Email** | **ORCID** | **Institution** |
| --- | --- | --- | --- |
| Andrea Barberis | andrea.barberis@galliera.it | 0000-0003-0472-0120 | Department of Abdominal Surgery - General and Hepatopancreatobiliary Surgery Unit, Ente Ospedaliero (E.O.) Galliera Hospital, Genova, Italy |
| Lorenzo Epis | lorenzo.epis@galliera.it | 0000-0001-7591-9335 | Department of Abdominal Surgery - General and Hepatopancreatobiliary Surgery Unit, Ente Ospedaliero (E.O.) Galliera Hospital, Genova, Italy |
| Marco Filauro | marco.filauro@galliera.it | 0000-0003-4029-400X | Department of Abdominal Surgery - General and Hepatopancreatobiliary Surgery Unit, Ente Ospedaliero (E.O.) Galliera Hospital, Genova, Italy |

| **First name and Last name** | **Email** | **ORCID** | **Institution** |
| --- | --- | --- | --- |
| Carolina Cecchi | cecchicarolina@gmail.com | 0000-0002-1222-439X | Hepatobiliary Surgery Unit, USL Toscana Centro, San Jacopo Hospital, Pistoia |
| Rosita De Vincenti | rosita.devincenti@uslcentro.toscana.it | 0000-0002-8474-8475 | Hepatobiliary Surgery Unit, USL Toscana Centro, San Jacopo Hospital, Pistoia |
| Massimo Fedi | massimo.fedi@uslcentro.toscana.it | 0000-0003-0386-9767 | Hepatobiliary Surgery Unit, USL Toscana Centro, San Jacopo Hospital, Pistoia |

| **First name and Last name** | **Email** | **ORCID** | **Institution** |
| --- | --- | --- | --- |
| Paolina Saullo | paolina.saullo@uniroma1.it | 0000-0003-3694-1761 | Department of Surgery, Sapienza University of Rome, Viale Regina Elena 324, 00161 Rome, Italy |

| **First name and Last name** | **Email** | **ORCID** | **Institution** |
| --- | --- | --- | --- |
| Massimiliano Fabozzi | massimiliano.fabozzi@ausl.re.it | 0000-0001-5102-0210 | Surgical Oncology Unit, Azienda Unità Sanitaria Locale-IRCCS di Reggio Emilia, Reggio Emilia, Italy |
| Andrea Morini | andrea.morini@ausl.re.it |  | Surgical Oncology Unit, Azienda Unità Sanitaria Locale-IRCCS di Reggio Emilia, Reggio Emilia, Italy |
| Maurizio Zizzo | maurizio.zizzo@ausl.re.it |  | Surgical Oncology Unit, Azienda Unità Sanitaria Locale-IRCCS di Reggio Emilia, Reggio Emilia, Italy |

| **First name and Last name** | **Email** | **ORCID** | **Institution** |
| --- | --- | --- | --- |
| Marco Giordano | marco.giordano@auslromagna.it |  | Surgical Department, Infermi Hospital, 47923 Rimini, Italy |
| Gianmarco Palini | palinigm@yahoo.it |  | Surgical Department, Infermi Hospital, 47923 Rimini, Italy |
| Luigi Veneroni | luigi.veneroni@auslromagna.it | 0000-0002-2037-2739 | Surgical Department, Infermi Hospital, 47923 Rimini, Italy |

| **First name and Last name** | **Email** | **ORCID** | **Institution** |
| --- | --- | --- | --- |
| Gian Luca Grazi | gianluca.grazi@unifi.it | 0000-0002-3279-0313 | HepatoBiliaryPancreatic Surgery, AOU Careggi, Department of Experimental and Clinical Medicine, University of Florence, Florence, Italy |
| Antonio Taddei | antonio.taddei@unifi.it | 0000-0003-2963-4085 | HepatoBiliaryPancreatic Surgery, AOU Careggi, Department of Experimental and Clinical Medicine, University of Florence, Florence, Italy |
| Luca Tirloni | luca.tirloni@unifi.it | 0000-0002-6060-2172 | HepatoBiliaryPancreatic Surgery, AOU Careggi, Department of Experimental and Clinical Medicine, University of Florence, Florence, Italy |

| **First name and Last name** | **Email** | **ORCID** | **Institution** |
| --- | --- | --- | --- |
| Gennaro Mazzarella | gennaromazzarella226@gmail.com |  | General Surgery, Sapienza University of Rome, Italy |

| **First name and Last name** | **Email** | **ORCID** | **Institution** |
| --- | --- | --- | --- |
| Giulia Bacchiocchi | giuliabacchiocchi96@gmail.com | 0000-0002-9755-6229 | Department of Surgical Science, University of Rome "Tor Vergata", 00133 Roma, Italy |

| **First name and Last name** | **Email** | **ORCID** | **Institution** |
| --- | --- | --- | --- |
| Edoardo Baldini | ebaldini@inwind.it | 0000-0002-0252-2190 | General Surgery Unit, Santa Maria delle Stelle Hospital, Melzo, Italy |

| **First name and Last name** | **Email** | **ORCID** | **Institution** |
| --- | --- | --- | --- |
| Alberto Brolese | alberto.brolese@apss.tn.it | 0000-0002-6362-9055 | Department of Surgery, Hepato-Biliary Surgery Unit, Santa Chiara Hospital, Trento, Italy |

| **First name and Last name** | **Email** | **ORCID** | **Institution** |
| --- | --- | --- | --- |
| Francesca Notte | francesca.notte@sabes.it | 0000-0002-3559-1782 | Department of General and Pediatric Surgery, Bolzano Central Hospital, Bolzano, Italy |
| Stefan Patauner | stefan.patauner@sabes.it | 0000-0001-7592-0934 | Department of General and Pediatric Surgery, Bolzano Central Hospital, Bolzano, Italy |
| Giovanni Scotton | giovanni.scotton@sabes.it | 0000-0003-2222-3822 | Department of General and Pediatric Surgery, Bolzano Central Hospital, Bolzano, Italy |

| **First name and Last name** | **Email** | **ORCID** | **Institution** |
| --- | --- | --- | --- |
| Riccardo Caruso | ricaruso2@gmail.com | 0000-0002-0953-2420 | General Surgery, HM Sanchinarro University Hospital, Health Sciences Faculty HM Hospitals, Camilo Jose Cela University, Madrid, Spain |
| Yolanda Quijano | yolandaquijanosigloxxi@gmail.com |  | General Surgery, HM Sanchinarro University Hospital, Health Sciences Faculty HM Hospitals, Camilo Jose Cela University, Madrid, Spain |
| Emilio Vicente | correo@emiliovicente.es |  | General Surgery, HM Sanchinarro University Hospital, Health Sciences Faculty HM Hospitals, Camilo Jose Cela University, Madrid, Spain |

| **First name and Last name** | **Email** | **ORCID** | **Institution** |
| --- | --- | --- | --- |
| Stefano Cantafio | stefano.cantafio@uslcentro.toscana.it |  | Department of Surgery, Santo Stefano Hospital, ASL Toscana Centro, Prato, Italy |
| Egidio Miranda | egidiomiranda@ymail.com |  | Department of Surgery, Santo Stefano Hospital, ASL Toscana Centro, Prato, Italy |

| **First name and Last name** | **Email** | **ORCID** | **Institution** |
| --- | --- | --- | --- |
| Federica Maffeis | fede.maffeis@yahoo.it | 0000-0002-2993-1381 | General Surgical Department St. Mary of Angels Hospital, Pordenone Azienda Sanitaria Friuli Occidentale, Pordenone, Italy |
| Paolo Ubiali | paolap57@alice.it | 0000-0002-9277-442X | General Surgical Department St. Mary of Angels Hospital, Pordenone Azienda Sanitaria Friuli Occidentale, Pordenone, Italy |
| Jaqueline Velkoski | jaquelinevelkoski89@gmail.com | 0000-0002-8147-6208 | General Surgical Department St. Mary of Angels Hospital, Pordenone Azienda Sanitaria Friuli Occidentale, Pordenone, Italy |

| **First name and Last name** | **Email** | **ORCID** | **Institution** |
| --- | --- | --- | --- |
| Francesco Tandoi | francesco.tandoi@uniba.it | 0000-0003-3166-6684 | AOU Consorziale Policlinico di Bari, Bari, Italy |

| **First name and Last name** | **Email** | **ORCID** | **Institution** |
| --- | --- | --- | --- |
| Alessandro Capozucco | alessandrocapozucco@live.it |  | General Surgery, Sapienza University of Rome, Italy |

| **First name and Last name** | **Email** | **ORCID** | **Institution** |
| --- | --- | --- | --- |
| Alessandro Liguori | dralesliguori@gmail.com | 0009-0007-7611-2534 | UOC Chirurgia Generale, AOU San Giovanni di Dio e Ruggi d'Aragona, 84131, Salerno, Italy |
| Alessandro Puzziello | apuzziello@unisa.it | 0000-0002-1970-7386 | UOC Chirurgia Generale, AOU San Giovanni di Dio e Ruggi d'Aragona, 84131, Salerno, Italy |
| Mariafelicia Valeriani | liciavaleriani8@hotmail.it | 0009-0009-5066-8096 | UOC Chirurgia Generale, AOU San Giovanni di Dio e Ruggi d'Aragona, 84131, Salerno, Italy |

| **First name and Last name** | **Email** | **ORCID** | **Institution** |
| --- | --- | --- | --- |
| Chiara Bettini | chiarabettini95@gmail.com | 0009-0004-6924-6292 | Department of Clinical and Experimental Sciences, Surgical Clinic, University of Brescia at UOC General Surgery 3, ASST Spedali Civili, Brescia |
| Martina Fricano | martinafricano@live.it | 0009-0002-1885-0559 | Department of Clinical and Experimental Sciences, Surgical Clinic, University of Brescia at UOC General Surgery 3, ASST Spedali Civili, Brescia |
| Sarah Molfino | sarahmolfino@gmail.com | 0000-0003-3510-9041 | Department of Clinical and Experimental Sciences, Surgical Clinic, University of Brescia at UOC General Surgery 3, ASST Spedali Civili, Brescia |

| **First name and Last name** | **Email** | **ORCID** | **Institution** |
| --- | --- | --- | --- |
| Giuseppe Evola | giuseppe_evola@hotmail.it | 0000-0002-3648-7063 | Department of General Surgery and Medical-Surgical Specialties, University of Catania, Catania, Italy |
| Luigi Piazza | lpiazza267@gmail.com | 0000-0002-6768-6962 | Department of General Surgery and Medical-Surgical Specialties, University of Catania, Catania, Italy |
| Marco Vacante | marcovacante@yahoo.it | 0000-0002-6815-5012 | Department of General Surgery and Medical-Surgical Specialties, University of Catania, Catania, Italy |

| **First name and Last name** | **Email** | **ORCID** | **Institution** |
| --- | --- | --- | --- |
| Alessandro Cucchetti | alessandro.cucchett2@unibo.it |  | Department of Medical and Surgical Sciences, University of Bologna, 40126 Bologna, Italy; General and Oncologic Surgery, Department of Surgery, Morgagni-Pierantoni Hospital, 47121 Forlì, Italy |
| Giorgio Ercolani | giorgio.ercolani2@unibo.it | 0000-0003-4334-5167 | Department of Medical and Surgical Sciences, University of Bologna, 40126 Bologna, Italy; General and Oncologic Surgery, Department of Surgery, Morgagni-Pierantoni Hospital, 47121 Forlì, Italy |
| Giuliano La Barba | giuliano.labarba@auslromagna.it |  | Department of Medical and Surgical Sciences, University of Bologna, 40126 Bologna, Italy; General and Oncologic Surgery, Department of Surgery, Morgagni-Pierantoni Hospital, 47121 Forlì, Italy |

| **First name and Last name** | **Email** | **ORCID** | **Institution** |
| --- | --- | --- | --- |
| Andrea Benedetti Cacciaguerra | dott.benedetti@gmail.com | 0000-0001-6886-8138 | Hepatobiliary and Abdominal Transplantation Surgery, Riuniti Hospital, Polytechnic University of Marche, Ancona, Italy |
| Federico Mocchegiani | federicomocchegiani@hotmail.com |  | Hepatobiliary and Abdominal Transplantation Surgery, Riuniti Hospital, Polytechnic University of Marche, Ancona, Italy |
| Marco Vivarelli | Vivarelli63@libero.it |  | Hepatobiliary and Abdominal Transplantation Surgery, Riuniti Hospital, Polytechnic University of Marche, Ancona, Italy |

| **First name and Last name** | **Email** | **ORCID** | **Institution** |
| --- | --- | --- | --- |
| Giammauro Berardi | Gberardi1@gmail.com | 0000-0001-8827-9189 | Division of General Surgery and Liver Transplantation, San Camillo Forlanini Hospital, Rome, Italy |
| Giuseppe Ettorre | Gmettorre@scamilloforlanini.rm.it |  | Division of General Surgery and Liver Transplantation, San Camillo Forlanini Hospital, Rome, Italy |

| **First name and Last name** | **Email** | **ORCID** | **Institution** |
| --- | --- | --- | --- |
| Alessandro Anselmo | alessandroanselmo.ptv@gmail.com | 0000-0003-1041-8120 | Minimally Invasive and Gastrointestinal Surgery Unit, Department of General Surgery, University of Rome Tor Vergata, Rome, Italy |
| Silvio Caringi | silvio.caringi@ptvonline.it |  | Minimally Invasive and Gastrointestinal Surgery Unit, Department of General Surgery, University of Rome Tor Vergata, Rome, Italy |
| Leandro Siragusa | leandros93@hotmail.it |  | Minimally Invasive and Gastrointestinal Surgery Unit, Department of General Surgery, University of Rome Tor Vergata, Rome, Italy |

| **First name and Last name** | **Email** | **ORCID** | **Institution** |
| --- | --- | --- | --- |
| Paolo Bianco | paolo.bianco@pinetagrande.it |  | Hepatobiliary and Pancreatic Surgery Unit, Pineta Grande Hospital, Castel Volturno, Caserta, Italy |
| Fulvio Calise | fulvio.calise@unimol.it |  | Hepatobiliary and Pancreatic Surgery Unit, Pineta Grande Hospital, Castel Volturno, Caserta, Italy |
| Salvatore Spiezia | salvatorespiezia00@gmail.com |  | Hepatobiliary and Pancreatic Surgery Unit, Pineta Grande Hospital, Castel Volturno, Caserta, Italy |

| **First name and Last name** | **Email** | **ORCID** | **Institution** |
| --- | --- | --- | --- |
| Alberto Patriti | albertopatriti@gmail.com | 0000-0003-2414-268X | General Surgery Unit, AST1, 61121 Pesaro, Italy |
| Filippo Petrelli | filippo.petrelli@sanita.marche.it |  | General Surgery Unit, AST1, 61121 Pesaro, Italy |

| **First name and Last name** | **Email** | **ORCID** | **Institution** |
| --- | --- | --- | --- |
| Luca Viganò | luca.vigano@gavazzeni.it | 0000-0002-4108-4832 | Hepatobiliary Unit, Department of Minimally Invasive General & Oncologic Surgery, Humanitas Gavazzeni University Hospital, Bergamo, Italy; Department of Biomedical Sciences, Humanitas University, Milan, Italy |

| **First name and Last name** | **Email** | **ORCID** | **Institution** |
| --- | --- | --- | --- |
| Antonio Giuliani | giuldoc@hotmail.com |  | UOC of General Surgery, San Giuseppe Moscati Hospital, Aversa, Italy |

| **First name and Last name** | **Email** | **ORCID** | **Institution** |
| --- | --- | --- | --- |
| Stefano Berti | stebe768@gmail.com |  | General Surgery Unit, Michele e Pietro Ferrero Hospital - Verduno - ASL CN2, Cuneo, Italy |
| Daniele Celi | dceli@aslcn2.it |  | General Surgery Unit, Michele e Pietro Ferrero Hospital - Verduno - ASL CN2, Cuneo, Italy |
| Valentina Marchese | vmarchese@aslcn2.it |  | General Surgery Unit, Michele e Pietro Ferrero Hospital - Verduno - ASL CN2, Cuneo, Italy |

| **First name and Last name** | **Email** | **ORCID** | **Institution** |
| --- | --- | --- | --- |
| Edoardo Maria Muttillo | edoardomaria.muttillo@uniroma1.it |  | Department of Medical Surgical Science and Translational Medicine, Sant'Andrea University Hospital, Sapienza University of Rome, Rome, Italy; Department of Digestive Surgery, Hopital Edouard Herriot, Lyon, France |
| Paolo Mercantini | paolo.mercantini@uniroma1.it |  | Department of Medical Surgical Science and Translational Medicine, Sant'Andrea University Hospital, Sapienza University of Rome, Rome, Italy; Department of Digestive Surgery, Hopital Edouard Herriot, Lyon, France |
| Andrea Scarinci | ascarinci@ospedalesantandrea.it |  | Department of Medical Surgical Science and Translational Medicine, Sant'Andrea University Hospital, Sapienza University of Rome, Rome, Italy; Department of Digestive Surgery, Hopital Edouard Herriot, Lyon, France |

| **First name and Last name** | **Email** | **ORCID** | **Institution** |
| --- | --- | --- | --- |
| Celia Caula | celiacaula@gmail.com | 0000-0002-5889-2414 | Hepatobiliary and Pancreatic Surgery Unit, Department of Surgery, Dr. Josep Trueta Hospital, IdIBGi, Girona, Spain |
| Margarida Casellas Robert | mcasellasro@gmail.com | 0000-0003-3349-5217 | Hepatobiliary and Pancreatic Surgery Unit, Department of Surgery, Dr. Josep Trueta Hospital, IdIBGi, Girona, Spain |
| Santiago Lopez Ben | santilb@msn.com | 0000-0002-5192-9966 | Hepatobiliary and Pancreatic Surgery Unit, Department of Surgery, Dr. Josep Trueta Hospital, IdIBGi, Girona, Spain |

| **First name and Last name** | **Email** | **ORCID** | **Institution** |
| --- | --- | --- | --- |
| Tommaso Campagnaro | tommaso.campagnaro@aovr.veneto.it | 0000-0001-7930-2455 | Division of General and Hepatobiliary Surgery, Department of Surgery, Dentistry, Gynaecology, and Paediatrics, University of Verona, University Hospital G.B. Rossi, Verona, Italy |
| Mario De Bellis | mario.debellis_01@univr.it | 0000-0001-7932-5921 | Division of General and Hepatobiliary Surgery, Department of Surgery, Dentistry, Gynaecology, and Paediatrics, University of Verona, University Hospital G.B. Rossi, Verona, Italy |
| Andrea Ruzzenente | andrea.ruzzenente@univr.it | 0000-0001-6911-563X | Division of General and Hepatobiliary Surgery, Department of Surgery, Dentistry, Gynaecology, and Paediatrics, University of Verona, University Hospital G.B. Rossi, Verona, Italy |

| **First name and Last name** | **Email** | **ORCID** | **Institution** |
| --- | --- | --- | --- |
| Roberto Montalti | robertomontalti@yahoo.it |  | Department of Clinical Medicine and Surgery, Division of HPB, Minimally Invasive and Robotic Surgery, Federico II University Hospital Naples, Naples, Italy |
| Gianluca Rompianesi | gianlucarompianesi@gmail.com | 0000-0003-0756-8013 | Department of Clinical Medicine and Surgery, Division of HPB, Minimally Invasive and Robotic Surgery, Federico II University Hospital Naples, Naples, Italy |
| Roberto Troisi | roberto.troisi@unina.it |  | Department of Clinical Medicine and Surgery, Division of HPB, Minimally Invasive and Robotic Surgery, Federico II University Hospital Naples, Naples, Italy |

| **First name and Last name** | **Email** | **ORCID** | **Institution** |
| --- | --- | --- | --- |
| Francesco Orlando | francesco.orlando@aocardarelli.it |  | Azienda Ospedaliera 'A. Cardarelli', Italy |
| Giovanni Vennarecci | giovanni.vennarecci@aocardarelli.it | 0000-0001-9727-4304 | Azienda Ospedaliera 'A. Cardarelli', Italy |

| **First name and Last name** | **Email** | **ORCID** | **Institution** |
| --- | --- | --- | --- |
| Francesco Ardito | francesco.ardito@unicatt.it |  | Hepatobiliary Surgery Unit, Foundation "Policlinico Universitario A. Gemelli", IRCCS, Catholic University, Rome, Italy |
| Francesco Razionale | francescorazionale@gmail.com |  | Hepatobiliary Surgery Unit, Foundation "Policlinico Universitario A. Gemelli", IRCCS, Catholic University, Rome, Italy |

| **First name and Last name** | **Email** | **ORCID** | **Institution** |
| --- | --- | --- | --- |
| Benedetto Ielpo | ielpo.b@gmail.com | 0000-0003-3129-3208 | Hepatopancreatobiliary Unit, Parc Salut Mar University Hospital, Barcelona, Spain |

| **First name and Last name** | **Email** | **ORCID** | **Institution** |
| --- | --- | --- | --- |
| Luca Risi | luca.risi@st.hunimed.eu | 0009-0000-5485-3728 | Department of Biomedical Sciences, Humanitas University, Milan, Italy |
